# A Flexible Proteomic Approach to A2 Bovine Milk Authentication Through Tryptic, Thermolytic, and Peptic Proteotypic Peptides Elucidated by Routine LC–MS Analysis

**DOI:** 10.1021/acs.jafc.6c04504

**Published:** 2026-05-19

**Authors:** Lorea R. Beldarrain, Miguel Ángel Sentandreu, Malen Sarasua, Leire Bravo-Lamas, Enrique Sentandreu

**Affiliations:** † Instituto de Agroquímica y Tecnología de Alimentos (IATA-CSIC), 46980 Paterna (Spain); ‡ Food Technology Department, Leartiker S. Coop., Leartiker S. Coop., 48270 Markina-Xemein (Spain)

**Keywords:** milk β-casein variants, milk fraud prevention, milk authentication, affordable proteomics, alternative proteases, routine quality assessment

## Abstract

The most common genotype for beta casein (β-CN) in European dairy cattle is A1A2, producing A1 and A2 variants merged in most traded bovine milks. A premium A2-milk category emerged worldwide through selection of A2A2-genotyped cows to produce added-value milk. Absence of A1 β-CN in bovine milk was assessed by a straightforward, cost-effective bottom-up low-resolution liquid chromatography–mass spectrometry (MS) methodology powered by ion-trap analysis. A preliminary data-dependent MS/MS exploratory approach characterized A1 and A2 variants in milk extracts digested with trypsin, pepsin, and thermolysin. Relative quantitation of selected peptide biomarkers was performed through Selected Reaction Monitoring analysis, detecting levels of 0.1% of A1 β-CN in milk mixtures from A1A1/A2A2-genotyped cows. Besides effective tryptic digestion, alternative thermolysin yielded short peptides that facilitated separation, while acidic pepsin supports the development of low-cost procedures. Affordability and robustness of this strategy favor its implementation by control laboratories addressing the authentication of A2 milk.

## Graphical abstract

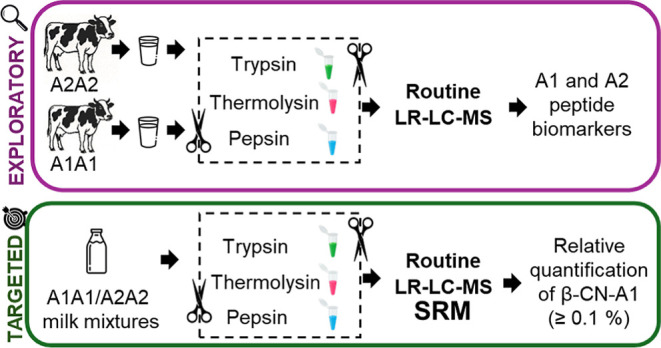

## 1 Introduction

Beta casein (β-CN) from bovine milk comprises 209 amino acid residues, registering 13 genetic variants because of single nucleotide polymorphisms in the CSN2 gene of animals. Predominant forms are A1 and A2 variants, differing only in one amino acid (Histidine and Proline in A1 and A2, respectively) at position 67 of the mature protein.^1^ Recent studies on the most common dairy cattle breed in Europe (Holstein-Friesian) evidenced that both A1 and A2 β-casein alleles are prevalent, with the A1 allele reaching frequencies ranging from 0.25 to 0.51 across European, American, and Australian populations, resulting in the simultaneous presence of both variants in most commercially available bovine milks.^2^ Consequently, the simultaneous presence of such variants is commonly found in traded European bovine milks.

Over the past two decades, the scientific community has showed a considerable interest in the physiological effects that the consumption of these two β-casein variants has on consumers. The presence of histidine in position 67 facilitates the liberation of β-casmorphin-7 (β-CM-7) during gastrointestinal activity of A1 β-CN, while the more enzymatically resistant proline–isoleucine bond of A2 variant favors the release of β-casmorphin-9 (β-CM-9) during digestion.^3^ Research showed that β-CM-7 had agonist opioid activity, showing affinity to the μ-opioid receptors in the gastrointestinal tract. It was suggested that β-CM-7 (hence, milk containing A1 β-CN) could cause negative effects in humans such as longer gastrointestinal transit times, digestive discomfort, and inflammation.^4^ Several recent studies suggested that for consumers who are not lactose intolerant or allergic to milk protein but experience gastrointestinal sensitivity consuming conventional milk, digestive discomfort could be relieved by consuming milk containing only the A2 variant of β-CN,^5,6^ although evidence is not yet conclusive. From this, farms worldwide aimed to convert their herds toward the production of A2 milk by selecting A2A2 genotyped animals. The popularity of milk and dairy products labeled as “A2 protein”, “A2 milk”, or “A2 β-CN” increased over the last years, boosting their consideration as innovative, premium, healthy products. Unambiguous authentication tools are then needed to ensure transparency for consumers,^7^ strengthening their confidence in the election of traded foodstuffs with enhanced nutritional or health-related attributes.

Cost-effective screening methods such as polymerase chain reaction (PCR) or enzyme-linked immunosorbent assay (ELISA) successfully addressed the study of A1 and A2 variants in bovine milk.^8,9^ Such solutions were commonly coupled to confirmatory qualitative/quantitative analyses supported by liquid chromatography–mass spectrometry (LC–MS) analysis.^10^ Current LC–MS proteomic workflows rely on the use of extremely efficient and budget-dependent high-resolution mass spectrometry (HRMS) technology for targeted/untargeted top-down^11^ and bottom-up^12–15^ qualitative and quantitative studies. In contrast, more limited but affordable targeted quantitative assays can be appropriately conducted by triple-quadrupole (QQQ) low-resolution mass spectrometry (LRMS) analyses. Targeted quantitative approaches featuring multiple reaction monitoring (MRM) facilitated by QQQ devices were considered to perform easier and are sensitive analysis of tryptic peptides.^16,17^ It must be emphasized that this MRM analysis lacks flexibility considering its inherent inability to perform the exploratory approach of samples addressing elucidation of potential peptide biomarkers. Thus, its usefulness is restricted to only targeted quantitative assays of peptides formerly characterized by an untargeted qualitative assay that includes interrogation of merged MS^1^ and MS/MS data against protein databases through search engines.

The development of flexible and economic LC–MS targeted/untargeted qualitative/quantitative proteomic pipelines easily implementable by the dairy industry for routine quality assessment of A1 and A2 bovine milks is needed. In this line, the exploration of β-CN protein fragmentation patterns other than that obtained by trypsin digestion becomes necessary, since the amino acid sequence surrounding β-CM-7 and β-CM-9 peptides contains several proline residues with no presence of arginine or lysine, thus giving rise to abnormally long tryptic peptides difficult to handle. Then, alternative fragmentation patterns such as those that can be obtained by thermolysin and pepsin must be considered. In addition, the case of pepsin is worth considering, taking into account the enhanced pepsin stability under acidic conditions and lower commercial costs (mainly compared to trypsin).

This study proposes a straightforward, flexible, and accessible proteomic methodology powered, for the first time, by conventional ion-trap (IT) analysis to create innovative proteomic insights for milk quality assessment. Affordability of IT technology is higher than that of current state-of-the-art LC-HRMS platforms, considering hardware use/maintenance and per-sample analysis costs (mainly if external service cores are required, finding higher fares in HRMS runs compared to LRMS). Despite the intrinsic technical limitations of IT analysis (mainly lower untargeted qualitative specificity and lower targeted quantitative sensitivity than HRMS and QQQ analyses, respectively), this simpler alternative can be of great interest to address bulk assays in routine control research.

Samples were first approached by an untargeted qualitative assay merging routine MS^1^-MS/MS data-dependent (dd-MS^2^) analysis to unveil key peptides discriminating A1 and A2 forms, followed by selected reaction monitoring (SRM) assays for targeted quantitation of selected candidates. Elucidation of discriminant A1 and A2 peptides was carried out on tryptic, thermolytic, and peptic digests to favor adaptation of this methodology to enzymatic arsenal available at control laboratories. The technical simplicity and robustness of this accessible approach would facilitate its implementation in routine quality assessment, ensuring consumer access to milk with verified compositional integrity.

## 2 Materials and Methods

### 2.1 Chemicals

LC–MS grade formic acid (FA), acetonitrile (ACN), and hydrochloric acid (HCl) were from Scharlab (Scharlab S.L., Barcelona, Spain). Ultrapure grade water was from Millipore (EMD Millipore Co., Billerica, MA, USA). Trypsin Gold (V5280) and pepsin (V1959) were from Promega (Promega Co., Madison, WI, USA). Thermolysin (P1512), sodium acetate, and ammonium bicarbonate were from Merck (Merck Life Sciences, Madrid, Spain). The Bradford Protein Assay Kit was from Bio-Rad (Bio-Rad, Hercules, CA, USA). Azidiol (176131.1611) was from PanReac AppliChem ITW Reagents (Panreac Química S.L.U., Barcelona, Spain).

### 2.2 Preparation of Internal Standards (IS) Solution

A wild almond protein extract was used as an IS, prepared as previously described,^18^ using tryptic digestion.

### 2.3 Milk Sampling

Raw milk was individually collected from six Friesian cows, yielded from morning milking (100 mL per animal) at a commercial dairy farm, adding azidiol (0.2%, v/v) as a preservative. Six animals were previously genotyped for the CSN2 gene (A1A1, *n* = 3; A2A2, *n* = 3) by the Genomic Service of the Spanish Holstein Confederation using the single nucleotide polymorphism (SNP) best linear unbiased prediction (BLUP) methodology.

Sampling procedure was schematized in Figure S1 to enhance clarity. Initially, milk samples assayed were divided into two groups (Figure S1A). The replicate group populating 6 samples (*n* = 3 for A1A1 and A2A2 genotypes, respectively) and the mixture group (*n* = 5) prepared by increasing volumes of spiked replicate 4 (A1A1) into replicate 1 (A2A2) sample, resulting in mixtures of A2 β-CN milk containing 0.1, 0.5, 1, 3, and 5% v/v of A1 β-CN milk.

### 2.4 Casein Handling for Peptide Releasing

To obtain skimmed milk, replicate and mixture samples described above were centrifuged at 6000 rpm for 30 min (Figure S1B). The intermediate layer was collected, and 0.1 M sodium acetate buffer (pH 4.6) was added in a 2:3 ratio (v/v). The mixture was centrifuged at 13000 rpm for 30 min, and the β-CN pellet was recovered after discarding the supernatant. The pellet was washed with 2 mL of ultrapure cold water, centrifuged at 13000 rpm for 10 min, and the supernatant was discarded. The washed β-CN pellet was finally redissolved in 1 mL of 50 mM ammonium bicarbonate (NH_4_HCO_3_) with sonication during 15 min, and protein concentration was determined through the Bradford assay.

Redissolved β-CN pellets from replicate and mixture groups were separately digested with mass spectrometry-grade trypsin, thermolysin, and pepsin solutions prepared according to their respective manufacturers’ instructions. Proteases were added to β-CN extracts at appropriate enzyme-to-substrate ratios according to the manufacturer’s instructions and incubated overnight at 37 °C with continuous shaking. Thus, a trypsin solution (20 μg/μL) was used at a 1:200 (w/w) ratio, meanwhile a thermolysin solution (1 μg/μL) was assayed at a 1:12.5 (w/w) ratio. Regarding the pepsin study, casein extracts were first acidified to pH 2.0 with 1 M HCl and subsequently digested through a 1 μg/μL enzyme solution added at a 1:66 (w/w) ratio. In all cases, SpeedVac-mediated desiccation was applied to all digested casein extracts that were finally resuspended in 300 μL of 0.1% FA aqueous solution.

### 2.5 Creation of LC–MS Batches

Creation of sample batches addressing LC–MS analyses was identical for all three enzymes assayed (Figure S1C). 35 μL of digested replicate samples were spiked with 15 μL of the IS solution, stirred, centrifuged (10000 rpm for 3 min) and transferred to LC–MS vials (REP batch, *n* = 6). A separate CAL batch (*n* = 6) was constructed by pooling 35 μL from each REP sample to give a final volume of 210 μL. From this, a calibration curve was constructed at different levels: 5, 10, 20, 30, 40, and 50 μL of the pooled sample were mixed with 15 μL of the IS solution and a variable volume of ultrapure water to reach a final volume of 65 μL. Samples were vortexed, centrifuged (13000 rpm for 3 min), and transferred to LC–MS vials. For technical quality assessment, a quality control (QC) sample was prepared by spiking 15 μL of IS solution into 70 μL of the aforementioned pooled sample, followed by stirring and centrifugation (12000 rpm for 3 min) and pouring into an LC–MS vial. This technical sample was repeatedly injected throughout the LC–MS injection sequence corresponding to REP batch analysis, constituting the QC batch (*n* = 3). Finally, 70 μL of MIX samples were spiked with 15 μL of the IS solution, stirred, centrifuged (13000 rpm for 3 min), and transferred to LC–MS vials (MIX batch, *n* = 5).

### 2.6 LC–MS Analyses

Analyses were performed in an Accela LC system coupled to an LTQ Velos Pro ion-trap (Thermo Sci., San Jose, CA, USA) mass spectrometer operating in the positive electrospray (ESI) ionization mode. Separation of peptides was achieved on a 150 mm × 2.1 mm × 3 μm Phenomenex Luna Omega PS C18 column (Phenomenex Inc., Torrance, CA, USA). First, an exploratory approach of samples exclusively containing A1 or A2 β-CN variants was conducted for the unambiguous characterization of proteotypic peptides that will be used in a secondary targeted SRM quantitative approach. From REP batches belonging to all three enzymes assayed (Figure S1C), samples 1 and 4, corresponding to A2 and A1 milks, respectively, were selected to carry out the preliminary qualitative approach. Chromatographic separation was carried out using ultrapure water/FA (99.9:0.1, v/v) as mobile phase A and ACN/FA (99.9:0.1, v/v) as mobile phase B. The separation gradient was initially 0% B held for 2 min, then linear 0–80% B for 23 min, washing at 95% B for 5 min, and column equilibration at 0% B for 15 min; the total run time was 45 min. The flow rate was 200 μL/min; the autosampler temperature was 10 °C; the column temperatures for tryptic/peptic and thermolytic digests were 23 and 50 °C, respectively; and the injection volumes for tryptic/peptic and thermolytic digests were 3 and 2 μL, respectively. Depth of this preliminary qualitative analysis was expanded through the inherent capacity of linear ion traps for merging Collision-Induced Dissociation (CID) and Higher-energy Collisional Dissociation (HCD) activation energies during data-dependent (dd-MS^2^) analyses carried out under the following operational conditions: capillary temperature, 300 °C; spray voltage, 4.0 kV; considered *m*/*z* range for MS^1^ analysis, 300–2000; sheath and auxiliary gases (arbitrary units), 45 and 10, respectively; default charge state of peptides for MS/MS analysis, +2; normalized CID and HCD energies, 35 and 30%, respectively; minimum MS/MS ion intensity threshold, 1 × 10^5^; exclusion list, 50 masses from spiked ISs and background noise from a blank injection; isolation width, 2 amu; exclusion mass width, 0.5 (low) and 1.2 (high); reject mass width, 0.5; exclusion time, 12 s; repeat count for MS/MS of most intense ion, 1; repeat count duration, 0.5 min; number of microscans–maximum injection time was 1–100 for MS^1^ and MS/MS experiments.

The initial exploratory approach allowed the elucidation of definitive SRM transitions under optimal activation energy conditions for targeted quantitative analysis (see Results and Discussion Section). In this late analytical step, the same aforementioned mobile phases were considered for chromatographic separation of tryptic, peptic, and thermolytic digests. In the case of tryptic and peptic samples, the same gradient and general chromatographic settings were used, with the only difference in the flow rate, which was adjusted to 150 μL/min for the SRM assay. Regarding quantitative analysis of thermolytic samples, chromatographic separation conditions were rather different from those described above: initially, 0% B was held for 2 min, then linear 0–42% B for 12 min, washing at 95% B for 6 min, and column equilibration at 0% B for 10 min; the total run time was 30 min. Flow rate and injection volume were 500 μL/min and 2 μL, respectively; autosampler and column temperatures were set at 10 and 50 °C, respectively.

The number of SRM fragments and events were defined according to considerations made by Fuente-García et al.^18^ to maximize reliability of generated quantitative data. Each SRM event consisted of the summation of 3 characteristic MS/MS fragments from respective precursor ions, limiting the number of events to a maximum of 10 (including those from ISs) according to experimental chromatographic peak widths (around 0.4 min as an average) and rapidity of the MS detector (1.8 s/scan), giving rise to a minimum of 14 scans across the peak. Operational settings of the mass analyzer featuring SRM analysis were as follows: capillary temperature, 300 °C (325 °C for thermolytic digests); spray voltage, 4.0 kV; sheath and auxiliary gases (arbitrary), 45 (55 for thermolytic digests) and 10 (15 for thermolytic digests), respectively; number of microscans and maximum injection time, 1–50 ms; normalized CID and HCD energies, 35 and 30%, respectively; and number of SRM events, 10 (tryptic and peptic samples) and 6 (thermolytic samples). In the case of SRM analyses ruled by HCD breakdown, the charge state of parent masses was also incorporated into the analytical method (determined by the preliminary exploratory approach) to enhance the sensitivity of detection.

Control of the LC–MS device was done by using a PC loaded with Thermo Xcalibur v2.04 software (Thermo Sci, San Jose, CA, USA).

### 2.7 Processing of Preliminary MS^1^ and dd-MS^2^ Qualitative Data

Structural MS/MS data from tryptic, thermolytic, and peptic A1 and A2 peptides was interrogated using the Mascot v3.1 (www.matrixscience.com) search engine loading UniprotKB and Swissprot protein databases. The false discovery rate threshold was set at 1%. For trypsin and pepsin digests, the respective enzymes were specified for Mascot research, whereas for thermolysin samples, the “None” option was selected for data interrogation. Assignments with a protein score derived from individual ion scores indicating identity or extensive homology (*P* ≤ 0.05) were considered as true protein identifications. Protein sequences used for variant identification were CASB_BOVIN and A0A452DHW7 from the SwissProt and UniprotKB databases. CASB_BOVIN corresponds to A2 variant of β-CN containing a proline at position 67 of the mature protein, whereas A0A452DHW7 has histidine at position 67 that characterizes the A1 variant, as well as an additional sequence modification at position 122. For practical reasons, this sequence was employed rather than the reference A1 β-CN sequence from NCBI (AAA30431.1), as the latter would significantly increase computation time. The modification at position 122 does not compromise peptide identification.

### 2.8 In Silico Digestion of β-CN Variants

Theoretical proteolytic digests from CASB_BOVIN and A0A452DHW7 sequences were performed in silico using PeptideCutter (ExPASy, Swiss Institute of Bioinformatics, https://web.expasy.org/peptide_cutter) freeware to predict cleavage sites and peptide fragments of β-CN A1 and A2 variants according to enzymes assayed. Complete digestion at every predicted cleavage site was assumed, and no sequence modifications were considered. Molecular weights of peptides were also calculated through PeptideMass (ExPASy, Swiss Institute of Bioinformatics) freeware.

### 2.9 Processing of Targeted SRM Quantitative Data

From the preliminary exploratory approach, a list of proteotypic peptides of A1 and A2 variants was configured according to the proteases assayed. To enhance the sensitivity of targeted SRM quantitative analysis, those peptides with higher MS^1^ response (precursor ion) and good chromatographic shape were selected.^18^ Then, manual inspection of their MS/MS breakdown patterns and respective Mascot analysis eased the selection of the three most intense fragment ions from selected precursors. Relative quantitation was normalized according to peak area ratios of SRM transitions of target peptides with ISs. Data processing was performed by freely available FreeStyle 1.8 SP2, as previously described.^18^

## 3 Results and Discussion

LC–MS files (mzML format) of preliminary exploratory dd-MS^2^ and targeted quantitative SRM analyses are freely available at https://digital.csic.es/handle/10261/410079.

### 3.1 Selection of Characteristic Precursor Ions and SRM Transitions

Table 1 summarizes results yielded by the in silico digestion of A1 and A2 β-CN variants with enzymes assayed, gathering characteristic peptides containing the polymorphic residue at position 67 of mature protein. To enhance clarity, Figure S2 is a schematic representation of such characteristic peptides alongside the mature protein sequence.

**Table 1:** Amino Acid Sequences Obtained in Silico After Tryptic, Thermolytic, and Peptic Digestion of A1 and A2 Variants of β-casein (β-CN)

Digestion enzyme	β-CN variant^a^	Amino acid sequence^b^	Neutral theoretical Mass [M]^c^
Trypsin	A2	IHPFAQTQSLVYPFPGPI**P**NSLPQNIPPLTQTPVVVPPFLQPEVMGVSK	5315.85
	A1	IHPFAQTQSLVYPFPGPI**H**NSLPQNIPPLTQTPVVVPPFLQPEVMGVSK	5355.85
Thermolysin	A2	VYPFPGPI**P**NSLPQNIPP	1946.03
	A1	I**H**NSLPQNIPP	1228.66
Pepsin	A2	VYPFPGPI**P**NSL	1299.69
	A1	VYPFPGPI**H**NSL	1339.69

a β-CN sequences used for the search were A0A452DHW7 (UniprotKB) for the A1 variant and CASB_BOVIN for the A2 variant (SwissProt).

b Characteristic peptides with polymorphic amino acids at position 67 of the mature protein.

c Monoisotopic Mass.

Untargeted dd-MS^2^ analyses (using CID and HCD activation energies) of selected REP samples (REP 1 and REP 4 corresponding to A2 and A1 milk variants, see LC–MS Analyses section) coupled to Mascot protein interrogation confirmed the presence of the characteristic peptides detailed in Table 1 in multiple charge states (data not shown). A comparison of CID and HCD fragmentation patterns of such biomarkers was performed to select the most sensitive and robust SRM alternative, and CID provided a more stable fragmentation with a higher abundance of preponderant MS/MS fragments (data not shown). From this point onward, discussions of SRM analysis will be limited to CID fragmentation conditions.

Manual inspection of MS/MS experimental data from parent ions of characteristic peptide biomarkers from A1 and A2 REP samples and their Mascot results (Figures S3–S5) allowed the creation of the SRM-CID library (Table S1) used for targeted quantitative research. The in-house database listed the three most abundant characterized fragments from respective parent masses under the different digestion conditions assayed.

### 3.2 Reliability of Targeted SRM Quantitative Analysis of A1 and A2 Peptide Biomarkers

The relative quantitation approach of REP, CAL, and MIX batches is summarized in Tables S2–S4, finding normalized results according to respective ISs (normalizer IS, Table S1) and enzymes assayed. Furthermore, robustness of SRM analysis was assessed through the reliability ratios achieved between the normalizer and quality ISs (Table S1) spiked in all sample batches. Deviation of IS reliability ratios yielded between batches belonging to the same enzymatic assay was up to 10% (Tables S2–S4), confirming reproducibility of SRM analysis at this level. A further technical quality assurance step was provided by QC batch analysis (Table S5) testing repeatability of normalized A1 and A2 results together with IS reliability ratios alongside the control injection sequence, giving deviations up to 11% considering all enzymatic conditions studied. In this line, Figure S6 depicts the linearity of the SRM assay regarding calibration curves from A1 and A2 characteristic precursors in CAL batch analyses (Tables S2–S4), finding in all cases a correlation coefficient (R^2^) greater than 0.98. Results clearly demonstrated the robustness of SRM measurements and confirmed the reliability of comparative determinations coming from the REP batch approach (Tables S2–S4). In all cases, REP samples containing the A1 variant of β-CN showed no SRM signal of its A2 counterpart and vice versa (Figures S7–S9).

Sensitivity and specificity of SRM analysis were assessed through the MIX batch approach consisting of increasing amounts of A1 milk into A2 milk samples. As Figure 1 shows, A1 β-CN was clearly detectable even at the 0.1% level, finding remarkable correlation coefficients (R^2^ > 0.97; Figure S10) independently of enzymes assayed. These findings evidenced the usefulness of this SRM methodology for control laboratories addressing routine milk certification/authentication activities.

**Figure 1:**
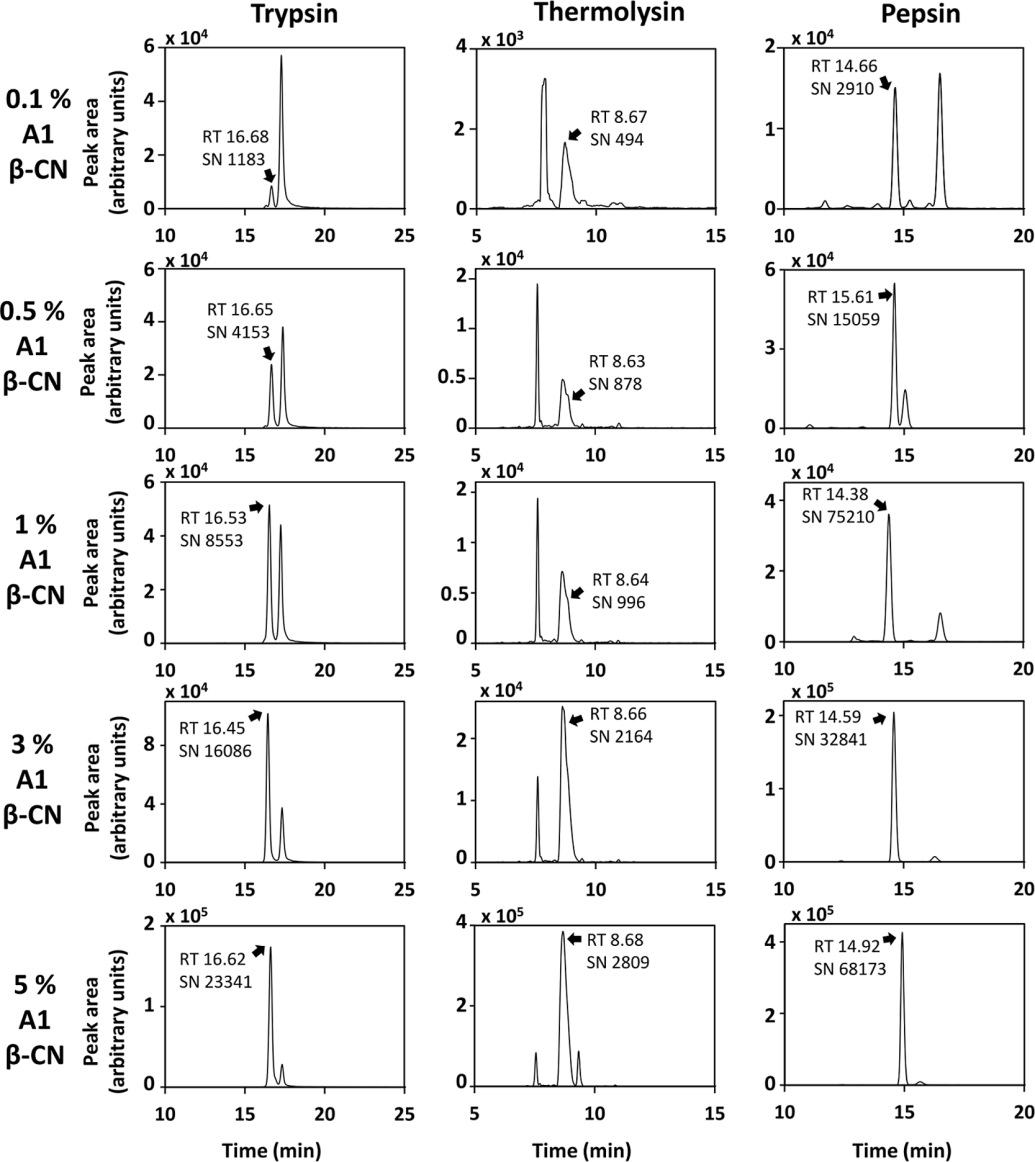
Chromatograms from targeted quantitative SRM analysis of trypsin, thermolysin, and pepsin MIX batches. Arrows indicate characteristic A1 precursors in samples assayed. Abbreviations used: CN, casein; RT, retention time; and SN, signal-to-noise ratio (from FreeStyle analysis, Section 2.9).

As a final confidence trial, Table S6 lists all reliability IS ratios yielded by REP, CAL, MIX, and QC batches from all enzymatic conditions assayed to demonstrate the technical robustness of the SRM assay. An overall deviation of 7–9% was achieved, being comparable to values achieved by previous HRMS analysis of A1 proteotypic peptides in milk,^13^ confirming reliability of this accessible methodology based on low-resolution LC–MS analysis.

### 3.3 Isomerization of Characteristic Thermolytic Peptides

Under tryptic and peptic chromatographic conditions (column temperature of 23 °C and flow rate of 150 μL/min), two adjacent peaks appeared in MS^1^ and SRM chromatograms of both A1 and A2 β-CN variants in thermolytic samples (Figure S11A, referred to MS^1^ of A1 variant in REP 4 sample). This can be understood considering that such biomarkers have two adjacent proline residues at the C terminus of their peptide sequences (see Table 1) and simultaneously occurring slow kinetics of isomerization during their chromatographic separation, as previously pointed out.^19^ The same authors demonstrated how more aggressive chromatographic conditions (column temperature of 50 °C and a 500 μL/min flow rate) successfully addressed the obtaining of one single quantifiable peak, as depicted by Figure S11B regarding MS^1^ of A1 variant in the REP 4 sample.

### 3.4 Applicability of the Proposed SRM Workflow for A1 β-CN Detection in A2 Milk

Several circumstances may lead to the presence of the A1 variant of β-CN in milk and dairy products stated as A2 products. That includes involuntary cross-contamination during milking or transportation, human error throughout the production chain, and/or intentional adulteration driven by the premium value of A2 milk in the market.^10^ Indeed, a recent survey conducted by Ehling et al.^16^ detected a 0.26–5.0% A1 β-CN range in commercial milk and milk-derived ingredients labeled as exclusive A2 products. In addition of having affordable and reliable tools enabling the genetic separation of milk-producing cows, the actual abundance of A1 β-CN in milk and dairy products should be accurately measured by control laboratories to satisfy the ethical trade currently demanded by consumers. Results achieved in this study (minimum detection level of A1 variant at 0.1% in milk, Figure 1) were comparable to those supported by advanced technology in raw milk,^12,16^ evidencing the usefulness of conventional ITs for routine quality assessment.

It should also be noted that targeted peptides studied are specific to the amino acid at position 67 of mature β-CN. Consequently, minor bovine β-CN variants carrying histidine at position 67 — namely B, C, F, and G — would be codetected as A1-type, while those carrying proline at this position — A3, D, E, H2, and I — would be codetected as A2-type. This is a biologically meaningful feature since all variants carrying histidine at position 67 are potential sources of BCM-7 upon gastrointestinal digestion and consistent with the food authentication objective proposed here. In practice, the contribution of minor variants in conventional milk analysis is expected to be negligible, as A1 and A2 are by far the most prevalent variants in European dairy cattle, finding variant B only in some breeds.

Regarding limitations, the present methodology was validated exclusively on raw bovine milk samples. Thermal processing treatments routinely applied in the dairy industry (mainly pasteurization and UHT sterilization) may affect casein structure and enzymatic digestibility through heat-induced modifications such as in Maillard reaction-mediated glycation of lysine residues, which could potentially block tryptic cleavage sites that can alter peptide recovery in bottom-up LC–MS workflows. However, previous results suggested that such interferences may not be critical under standard pasteurization conditions. Guo et al. (2022) specifically investigated tryptic A1 and A2 β-CN biomarker peptides in pasteurized milk by LC–MS/MS analysis, finding no evidence of glycation at their lysine residues.^17^ These findings suggest that tryptic bottom-up approaches for β-CN variant detection may be robust to pasteurization-induced modifications. Nevertheless, more severe thermal treatments such as UHT sterilization and other dairy matrices such as cheese, yoghurt, or infant formula will be assessed in future work by the presented SRM methodology for its broader implementation in routine milk control.

### 3.5 Elucidation of Alternative Enzymes to Determine A1 β-CN in A2Milk

Bottom-up proteomics was traditionally ruled by trypsin digestion, mainly considering its high specificity, availability, and easy handling. However, since exclusive reliance on this enzyme may limit the scope of a wide range of analytical approaches, the proteomics community recently started exploring alternative proteases.^20^ This partially moved forward the current study toward the consideration of alternative peptic and thermolytic digests aiming at the elucidation of reliable milk biomarkers.

As reported by Liu et al.,^13^ thermolysin digestion generates A1 and A2 peptides (with different lengths, Table 1) containing the polymorphic amino acid that facilitates their chromatographic separation. A shorter length of thermolytic peptides compared to their tryptic counterparts enhances their MS sensitivity since basic sites and length of peptides are positively correlated with the number of charge states in ESI ionization.^21^ Pepsin digestion exhibits similar advantages through the generation of 12 amino acid length peptides with charges of 2+ or lower, as shown by the selected peptic precursors assayed (Table S1).

It must be noted that proteolysis by sequencing-grade trypsin is a budget-dependent procedure considering its refined biotechnological design to provide an increased specificity with a lower autolytic rate. Despite its claimed gold standard consideration in proteomics research, the activity of trypsin can be hindered when facing hydrophobic and tightly folded proteins,^22^ requiring extreme conditions to favor exposure of their cleavage sites (expanding their sequence coverage by LC–MS analysis). Additional advantages of pepsin’s acidic and thermolysin’s thermal stabilities are the inactivation of contaminant enzymes and/or the prevention of microbial growth in their respective assayed media.

All these alternative enzymatic strategies complement bottom-up proteomic approaches traditionally supported by tryptic digestion, enhancing the robustness and flexibility of this SRM methodology that can be easily adapted to different technical requirements and available resources of control laboratories.

In conclusion, robustness, flexibility, and affordability of this SRM alternative can be of great interest to control laboratories addressing routine quality assessment of A1 β-CN-free milk, favoring all actions aimed at the improvement of commercial transparency and consumers’ trust. In further studies, the development of an analogous methodology but applied to processed dairy products would extend the applicability of this methodology to a wider range of protein foodstuffs.

## Supplementary Material



## Notes

Schematic representation of the sampling procedure, including milk sampling, casein handling, and SRM batching; schematic in silico representation of characteristic peptides containing the polymorphic residue in β-CN variants; MS/MS Mascot results from tryptic, thermolytic, and peptic digests; SRM linearity in calibration batches; SRM results from REP batch analyses; SRM linearity in mixture batches; MS^1^ chromatograms of the thermolytic A1 β-CN variant under different chromatographic conditions; SRM-CID transition library; targeted SRM quantitative results for tryptic, thermolytic, and peptic batches; QC batch analysis; and summary of IS reliability ratios (PDF)

## Notes

This research did not involve human or animal participants.

## Abbreviations USED:

ACN: acetonitrile
β-CN: beta-casein
β-CM-7: β-casmorphin-7
β-CM-9: β-casmorphin-9
BLUP: best linear unbiased prediction
CAL: calibration
CID: collision-induced dissociation
dd-MS^2^: data-dependent MS/MS
ELISA: enzyme-linked immunosorbent assay
ESI: electrospray ionization
FA: formic acid
HCD: higher-energy collisional dissociation
HCl: hydrochloric acid
HRMS: high mass resolution
IS: internal standard
LC-MS: liquid chromatography–mass spectrometry
LRMS: low-resolution mass spectrometry
MIX: mixture
MRM: multiple reaction monitoring
PCR: polymerase chain reaction;
QC: quality control
QQQ: triple-quadrupole
REP: replicate
SNP: single nucleotide polymorphism
SRM: selected reaction monitoring.
