# Supplementary material for: A Flexible Proteomic Approach to A2 Bovine Milk Authentication Through Tryptic, Thermolytic, and Peptic Proteotypic Peptides Elucidated by Routine LC–MS Analysis
Source: J Agric Food Chem. 2026 May 19;74(21):16712–8. doi: 10.1021/acs.jafc.6c04504 (PMC13237760; doi:10.1021/acs.jafc.6c04504)
Supplement: Supplementary file 1 [file jf6c04504_si_001.pdf]

Supporting information

A flexible proteomic approach to A2 bovine milk authentication through tryptic, thermolytic and peptic proteotypic peptides elucidated by routine LC-MS analysis

Lorea R. Beldarrain*, Miguel Ángel Sentandreu, Malen Sarasua, Leire Bravo-Lamas, and Enrique Sentandreu

Table of contents:

Figure S1 - Schematic representation of the sampling procedure including milk sampling, casein handling and SRM batching

Figure S2 - Schematic in silico representation of characteristic peptides containing the polymorphic residue in β-CN variants

Figures S3-S5 - MS/MS Mascot results from tryptic, thermolytic and peptic digests

Figure S6 - SRM linearity in calibration batches

Figures S7-S9 - SRM results from REP batch analyses

Figure S10 - SRM linearity in mixture batches

Figure S11 - MS^1 chromatograms of thermolytic A1 β-CN variant under different chromatographic conditions

Table S1 - SRM-CID transition library

Tables S2-S4 - targeted SRM quantitative results for tryptic, thermolytic and peptic batches

Table S5 - QC batch analysis

Table S6 - Summary of IS reliability ratios

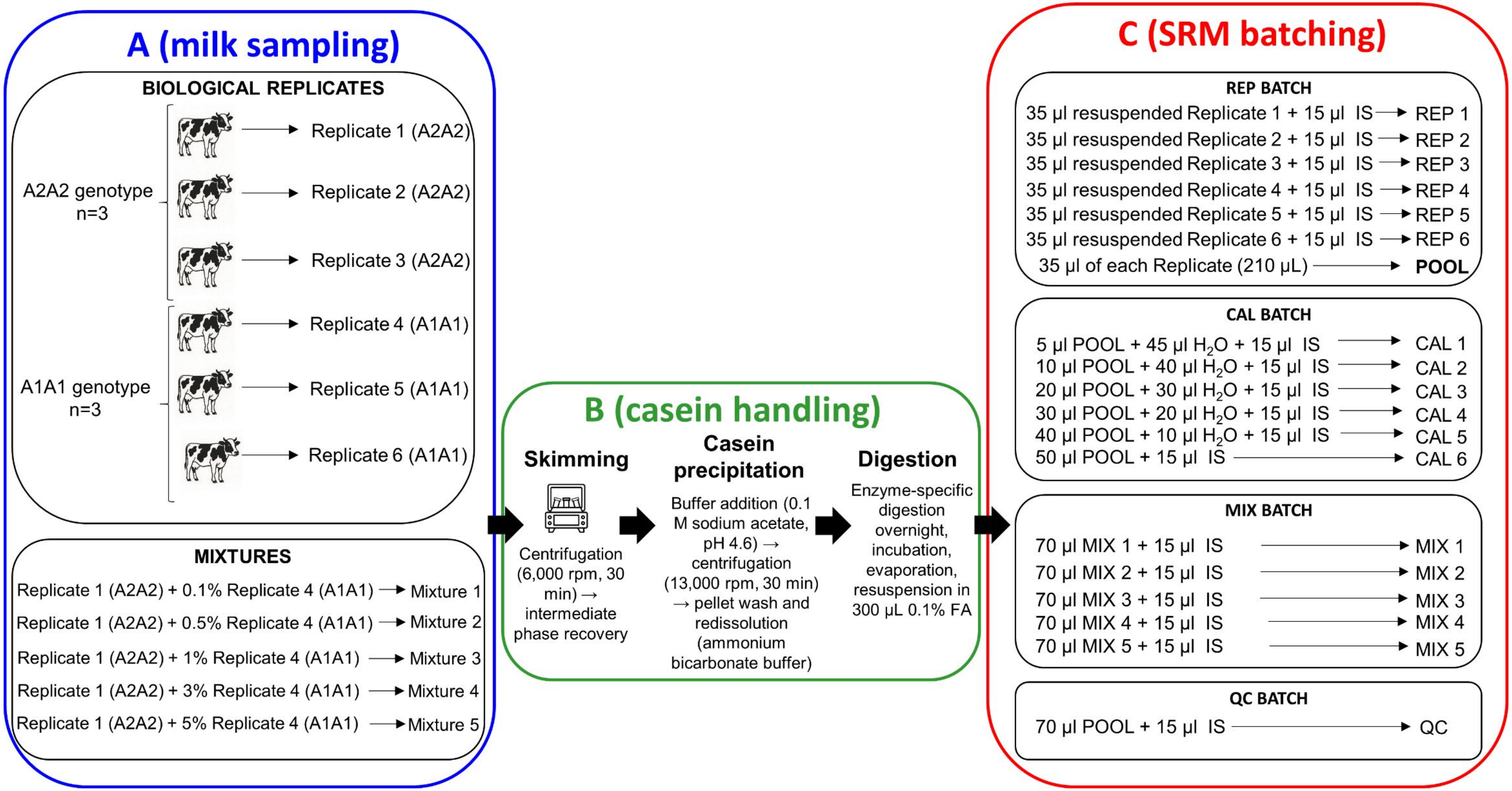

Figure S1: Schematic representation of the sampling procedure, including milk sampling (A), casein handling (B) and SRM batching (C). IS, internal standard; SRM, single reaction monitoring. CAL, calibration; MIX, mixture; REP: Replicate.

A

```
             1                                                50
A2 β-CN  RELEELNVPGEIVESLSSSEESITRINKKIEKFQSEEQQQTEDELQDKIHPFAQTQSLVYPFPGPI
             1                                                50
A1 β-CN  RELEELNVPGEIVESLSSSEESITRINKKIEKFQSEEQQQTEDELQDKIHPFAQTQSLVYPFPGPI
         *                                       100
A2 β-CN  PNSLPQNIPPLTQTPVVVPPFLQPEVMGVSKVKEAMAPKHKEMPFPKYPVEPFTESQSLTLT
         *                                       100                     *
A1 β-CN  HNSLPQNIPPLTQTPVVVPPFLQPEVMGVSKVKEAMAPKHKEMPFPKYPVEPFTERQSLTLT
                            150
A2 β-CN  DVENLHLPLPLLQSWMHQPHQPLPPTVMFPPQSVLSLSQSKVLPVPQKAVPYPQRDMPIQ
                            150
A1 β-CN  DVENLHLPLPLLQSWMHQPHQPLPPTVMFPPQSVLSLSQSKVLPVPQKAVPYPQRDMPIQ
                    200
A2 β-CN  AFLLYQEPVLGPVRGPFPIIV
                    200
A1 β-CN  AFLLYQEPVLGPVRGPFPIIV
```

B

```
             1                                                50
A2 β-CN  RELEELNVPGEIVESLSSSEESITRINKKIEKFQSEEQQQTEDELQDKIHPFAQTQSLVYPFPGPI
             1                                                50
A1 β-CN  RELEELNVPGEIVESLSSSEESITRINKKIEKFQSEEQQQTEDELQDKIHPFAQTQSLVYPFPGPI
         *                                       100
A2 β-CN  PNSLPQNIPPLTQTPVVVPPFLQPEVMGVSKVKEAMAPKHKEMPFPKYPVEPFTESQSLTLT
         *                                       100                     *
A1 β-CN  HNSLPQNIPPLTQTPVVVPPFLQPEVMGVSKVKEAMAPKHKEMPFPKYPVEPFTERQSLTLT
                            150
A2 β-CN  DVENLHLPLPLLQSWMHQPHQPLPPTVMFPPQSVLSLSQSKVLPVPQKAVPYPQRDMPIQ
                            150
A1 β-CN  DVENLHLPLPLLQSWMHQPHQPLPPTVMFPPQSVLSLSQSKVLPVPQKAVPYPQRDMPIQ
                    200
A2 β-CN  AFLLYQEPVLGPVRGPFPIIV
                    200
A1 β-CN  AFLLYQEPVLGPVRGPFPIIV
```

C

```
             1                                                50
A2 β-CN  RELEELNVPGEIVESLSSSEESITRINKKIEKFQSEEQQQTEDELQDKIHPFAQTQSLVYPFPGPI
             1                                                50
A1 β-CN  RELEELNVPGEIVESLSSSEESITRINKKIEKFQSEEQQQTEDELQDKIHPFAQTQSLVYPFPGPI
         *                                       100
A2 β-CN  PNSLPQNIPPLTQTPVVVPPFLQPEVMGVSKVKEAMAPKHKEMPFPKYPVEPFTESQSLTLT
         *                                       100                     *
A1 β-CN  HNSLPQNIPPLTQTPVVVPPFLQPEVMGVSKVKEAMAPKHKEMPFPKYPVEPFTERQSLTLT
                            150
A2 β-CN  DVENLHLPLPLLQSWMHQPHQPLPPTVMFPPQSVLSLSQSKVLPVPQKAVPYPQRDMPIQ
                            150
A1 β-CN  DVENLHLPLPLLQSWMHQPHQPLPPTVMFPPQSVLSLSQSKVLPVPQKAVPYPQRDMPIQ
                    200
A2 β-CN  AFLLYQEPVLGPVRGPFPIIV
                    200
A1 β-CN  AFLLYQEPVLGPVRGPFPIIV
```

Figure S2: Schematic *in silico* representation of characteristic peptides (highlighted in yellow) containing the polymorphic residue at position 67 (pointed out by an asterisk) of mature A2 and A1 variants of β-casein (β-CN) digested with trypsin (A), thermolysin (B) and pepsin (C). Protein sequences considered included CASB_BOVIN (Swissprot) and A0A452DHW7 (Uniprot KB), corresponding the former to A2 variant of β-CN with a proline at position 67 of mature protein, whereas the latter had histidine at position 67 and characterized the A1 form.

A

MS/MS Fragmentation of IHPFAQTQSLVYPFPGPIHNSLPQNIPPLTQTPVVVPPFLQPEVMGVSK
Found in A0A452DHW7 in UP9136_B_taurus, Beta-casein OS=Bos taurus OX=9913 GN=CSN2 PE=1 SV=1

Match to Query 3743: 5354.695120 from(1071.946300,5+) intensity(9366.4727) scans(6038) rawscans(sn6038) rtinseconds(947.8437
Title: 840: Scan 6038 (rt=947.844) [C:\TPP\data\Caseinas_leche24\Tanda 5 - Recorte 4_3_25\2.raw]
Local Instrument: ESI-TRAP
Data file Peak collection M2T5 no peak picking 3 to 5.mgf

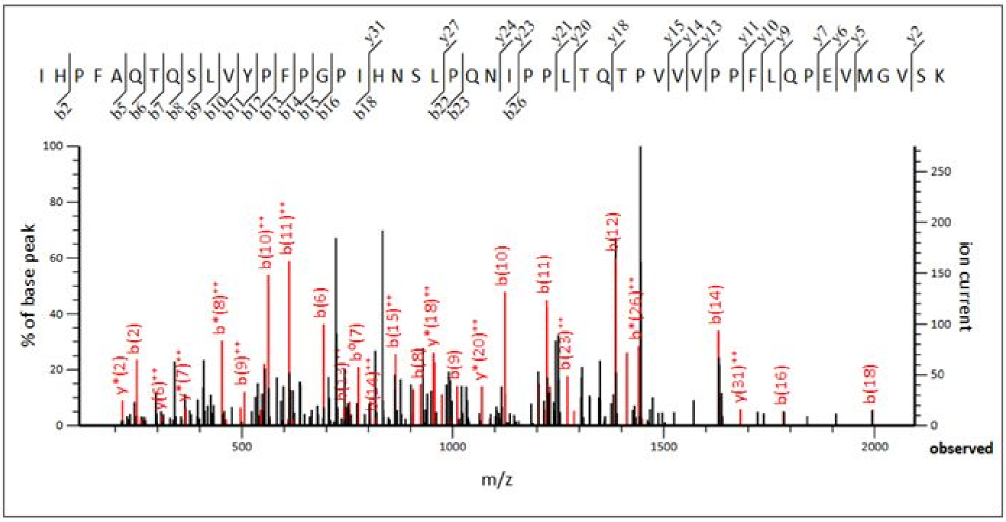

B

MS/MS Fragmentation of IHPFAQTQSLVYPFPGPIPNSLPQNIPPLTQTPVVVPPFLQPEVMGVSK
Found in CASB_BOVIN in SwissProt, Beta-casein OS=Bos taurus OX=9913 GN=CSN2 PE=1 SV=2

Match to Query 4201: 5316.788120 from(1064.364900,5+) intensity(83175.3980) scans(6573) rawscans(sn6573) rtinseconds(1002.9733) index
Title: 1329: Scan 6573 (rt=1002.97) [C:\TPP\data\Caseinas_leche24\Tanda 4 - Recorte 8_1_25\6-Copy.raw]
Local Instrument: ESI-TRAP
Data file M6T4 no peak pick 3 to 5.mgf

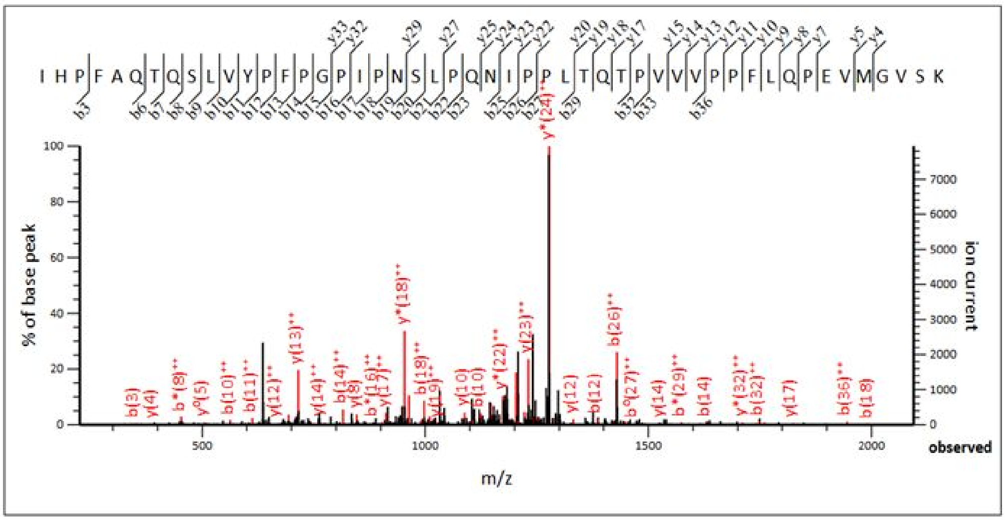

Figure S3: MS/MS Mascot results from the interrogation of exploratory dd-MS[2] CID analysis of selected trypsin-digested REP samples (see section 2.6) showing the positive assignment of characteristic A1-A0A452DHW7 (A) and A2-CASB_BOVIN (B) β-casein variants.

A

MS/MS Fragmentation of **IHNSLPQNIPP**
Found in **A0A452DHW7** in **UP9136_B_taurus**, Beta-casein OS=Bos taurus OX=9913 GN=CSN2 PE=1 SV=1

Match to Query 10569: 1229.197362 from(615.605957,2+) intensity(9640984.6562) scans(5785) rtinseconds(730.16) index(5558)
Title: T131.05785.05785.2
Data file T131.mgf

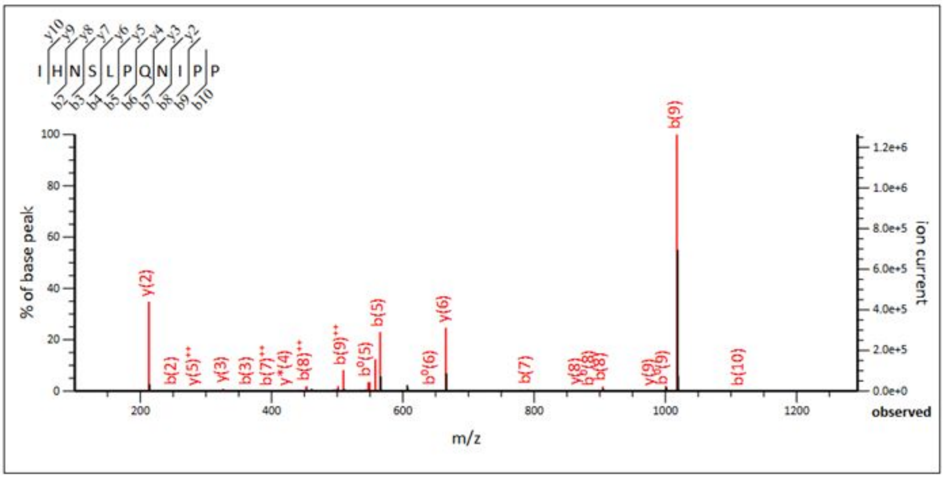

B

MS/MS Fragmentation of **VYPFPGPIPNSLPQNIPP**
Found in **CASB_BOVIN** in **SwissProt**, Beta-casein OS=Bos taurus OX=9913 GN=CSN2 PE=1 SV=2

Match to Query 247: 1946.686620 from(974.350586,2+) intensity(7282078.5) scans(5555) rtinseconds(754.28) index(126)
Title: Targeted974.05555.05555.2
Data file Targeted974.mgf

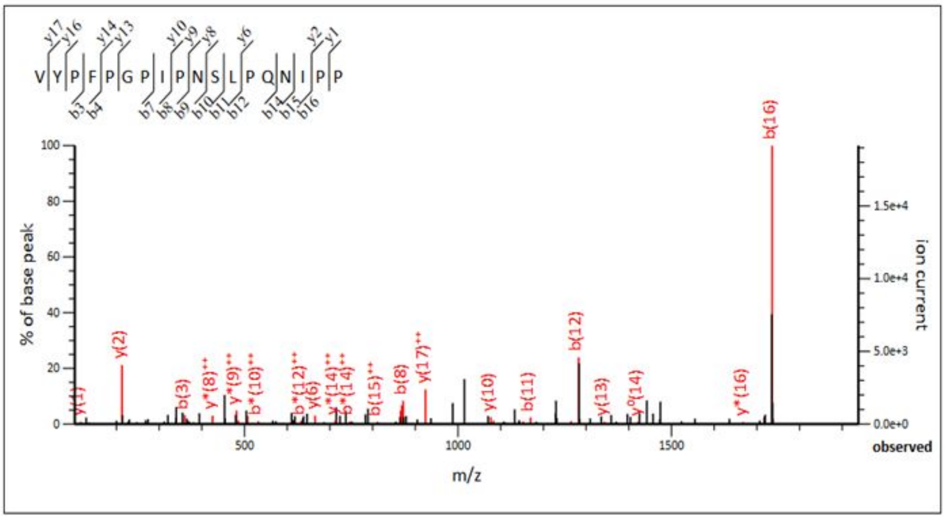

Figure S4: MS/MS Mascot results from the interrogation of exploratory dd-MS2 analysis of selected thermolysin-digested REP samples (see section 2.6) showing the positive assignment of characteristic A1-A0A452DHW7 (A) and A2-CASB_BOVIN (B) β-casein variants.

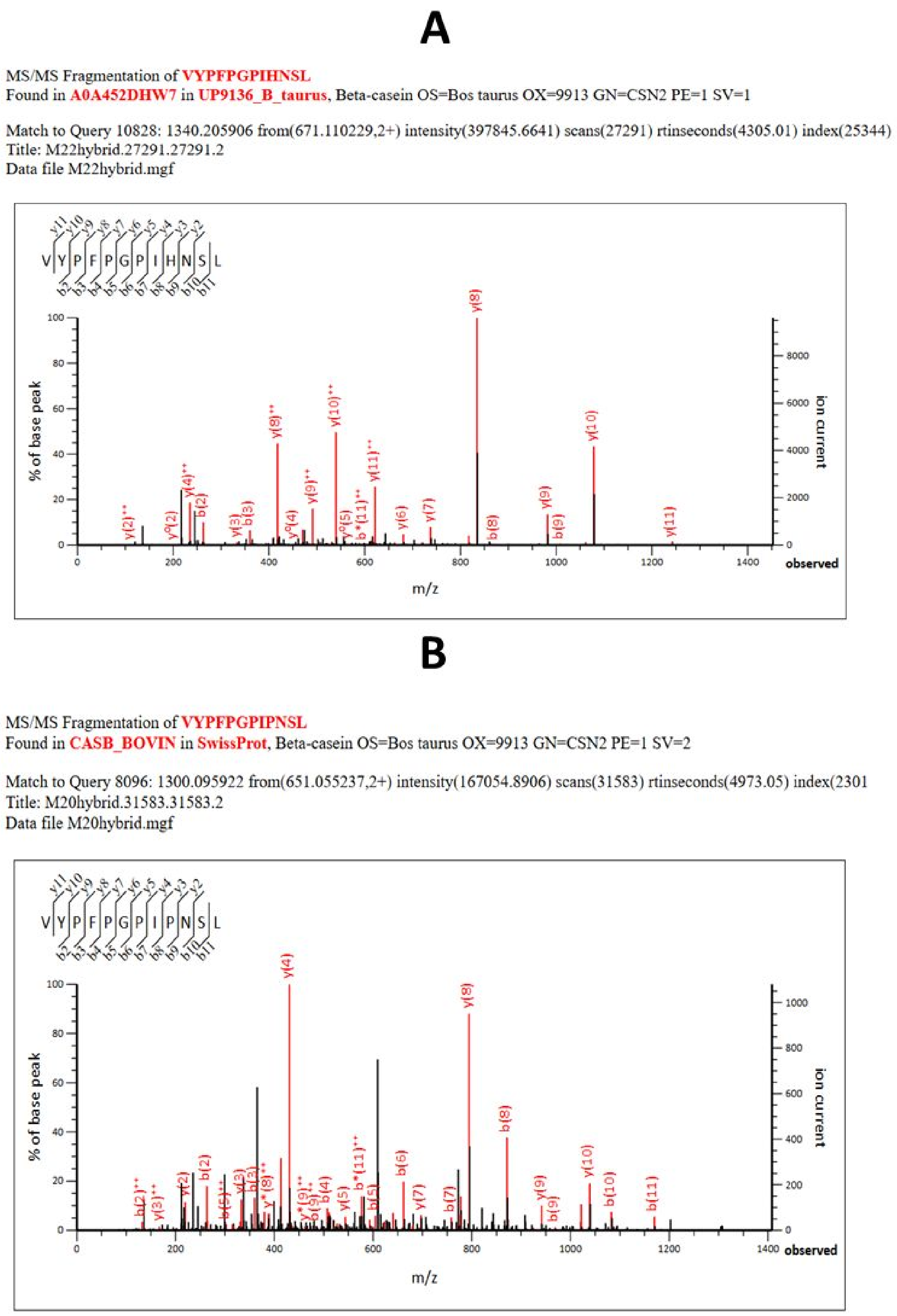

Figure S5: MS/MS Mascot results from the interrogation of exploratory dd-MS2 analysis of selected pepsin-digested REP samples (see section 2.6) showing the positive assignment of characteristic A1-A0A452DHW7 (A) and A2-CASB_BOVIN (B) β-casein variants.

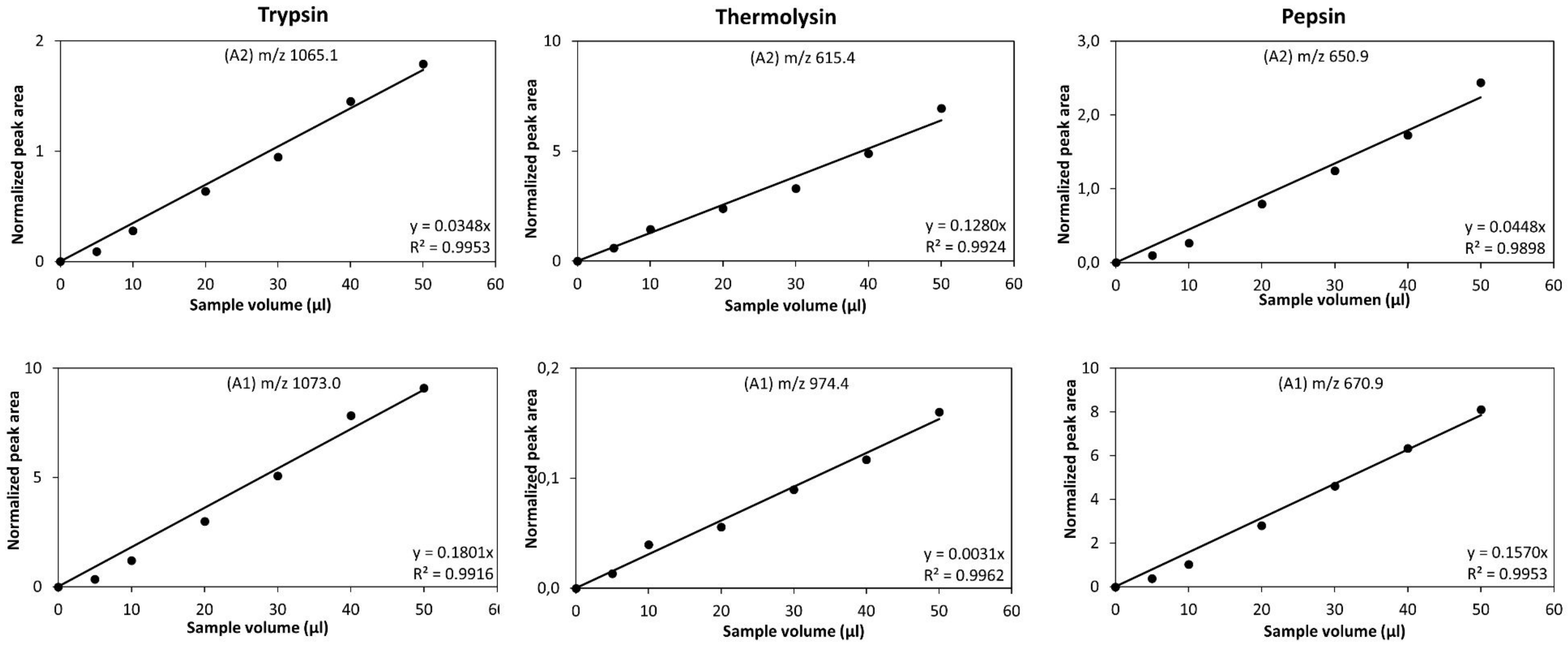

Figure S6: SRM linearity achieved in the calibration (CAL) sample batch for the characteristic precursor ions of A1 and A2 β-CN variants from tryptic, thermolytic and peptic digestions.

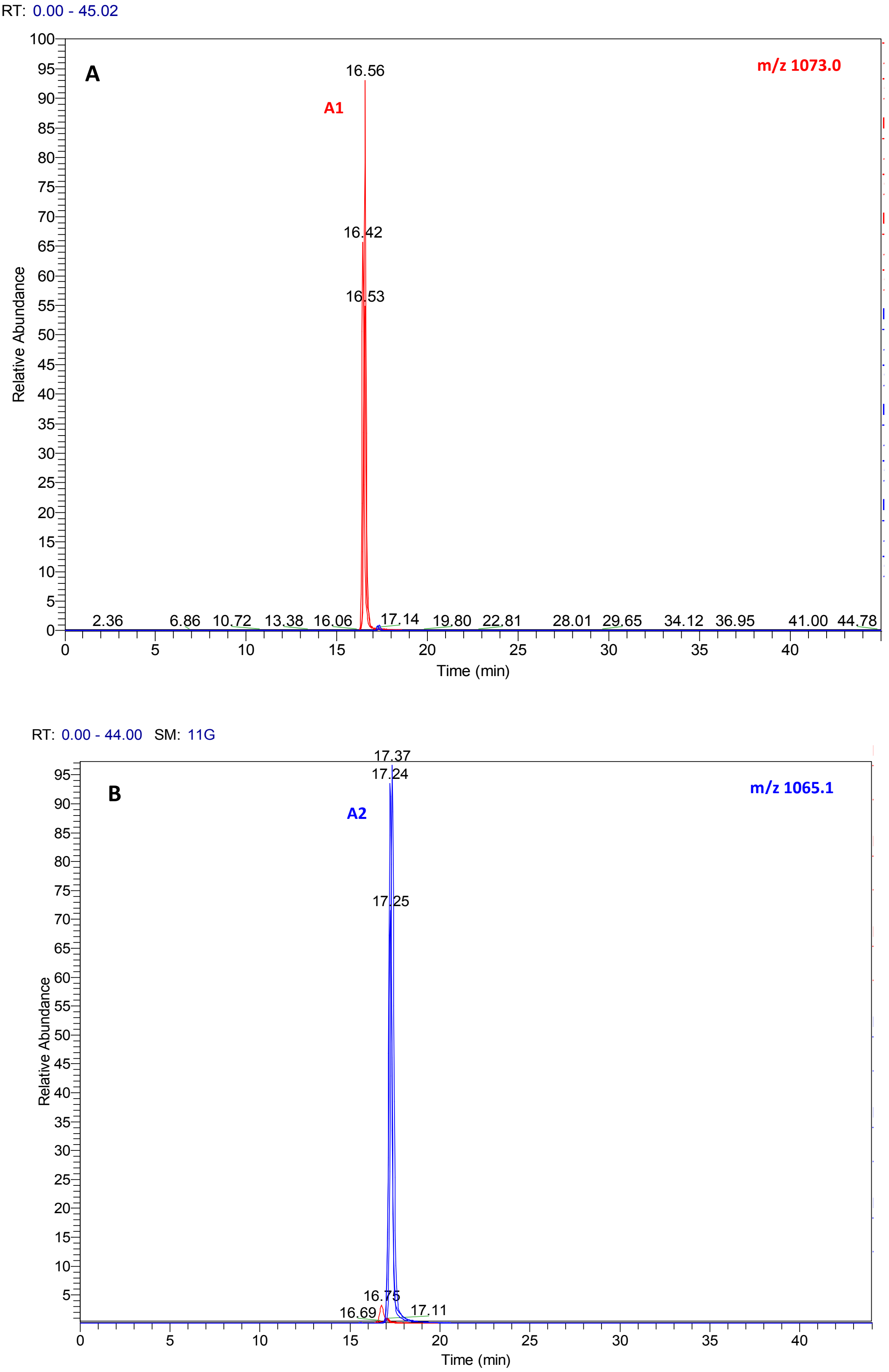

Figure S7: SRM results from REP batch analysis (samples 1-3, red; samples 4-6, blue) considering tryptic precursor ions at: m/z 1073.0 (A1 variant), (A); 1065.1 (A2 variant), (B). Sample characterization and SRM quantitative results of tryptic REP batch detailed in **Figure S1** and **Table S2**.

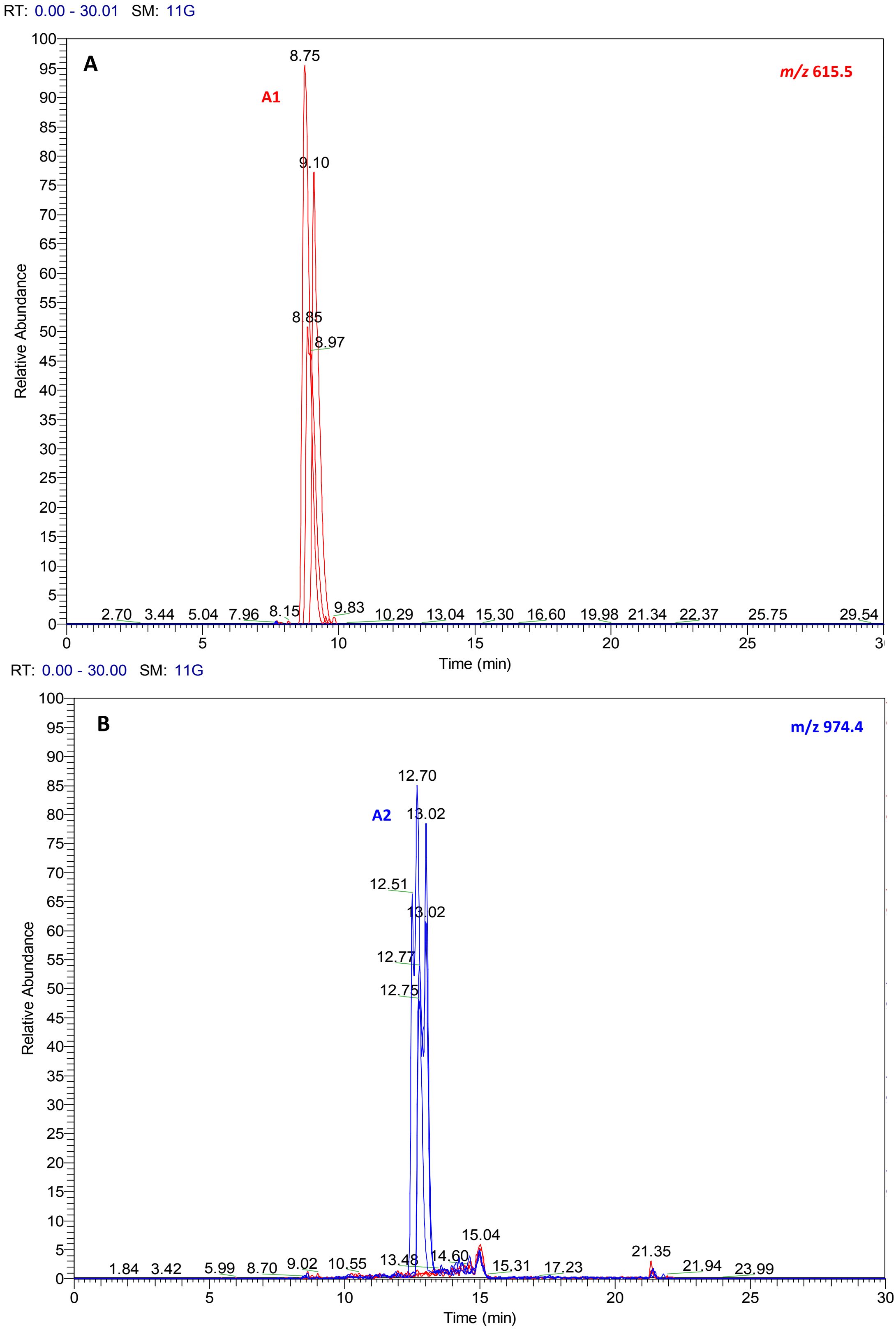

Figure S8: SRM results from REP batch analysis (samples 1-3, red; samples 4-6, blue) considering thermolytic precursor ions at: m/z 615.5 (A1 variant), (A); 974.4 (A2 variant), (B). Sample characterization and SRM quantitative results of thermolytic REP batch detailed in **Figure S1** and **Table S3.**

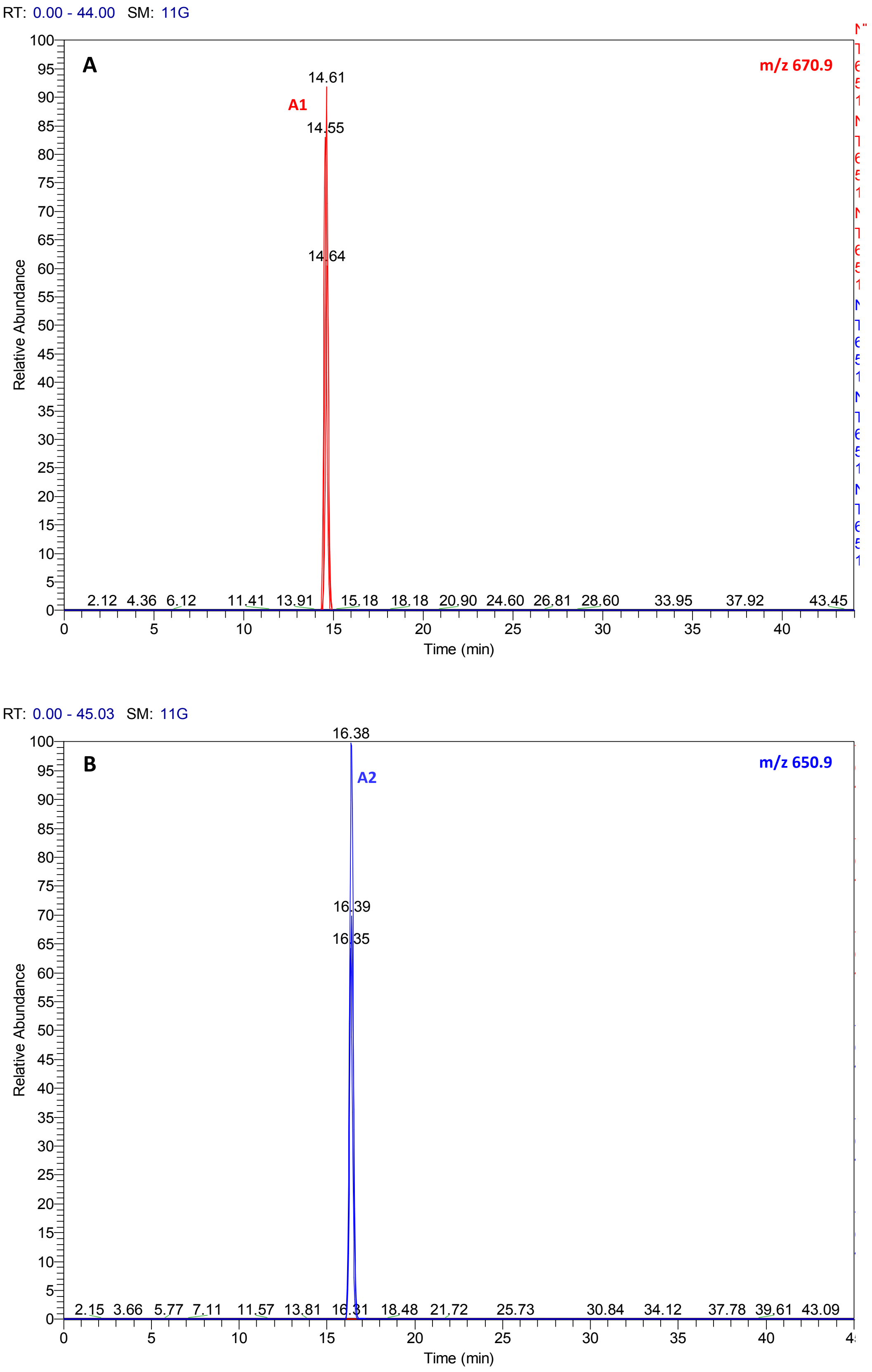

Figure S9: SRM results from REP batch analysis (samples 1-3, red; samples 4-6, blue) considering peptic precursor ions at: m/z 670.9 (A1 variant), (A); 650.9 (A2 variant) (B). Sample characterization and SRM quantitative results of thermolytic REP batch detailed in **Figure S1** and **Table S4.**

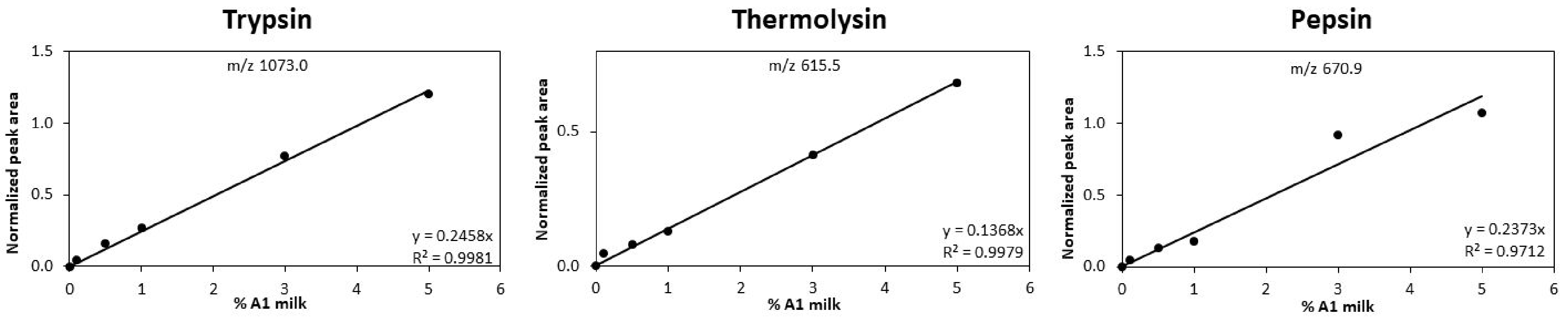

Figure S10: SRM Linearity achieved in the mixture (MIX) sample batch for the characteristic precursor ions of A1 β-CN variant obtained from tryptic, thermolytic, and peptic digestions.

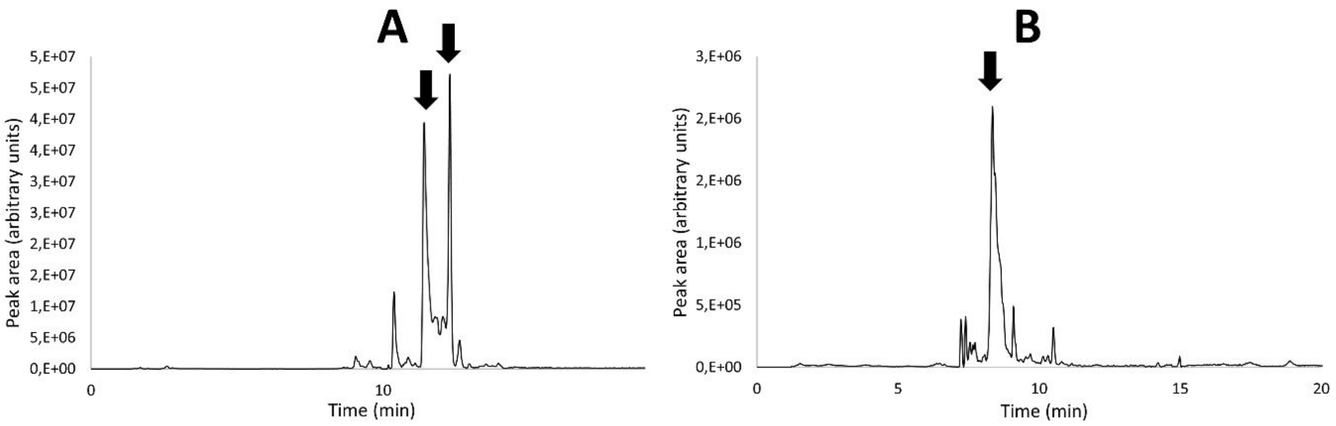

Figure S11: MS^1 chromatograms of A1 β-CN variant at *m/z* 615.5 in exploratory dd-MS^2 analysis of REP 4 sample digested with thermolysin. Chromatographic conditions were: A, column temperature and flow-rate of 23 °C and 150 µl/min, respectively; B, column temperature and flow-rate of 50 °C and 500 µl/min, respectively. Arrows indicate chromatographic peaks belonging to the observed parent ion.

Table S1. SRM-CID properties of characteristic A1 and A2 precursor ions (Table 1) under different chromatographic and enzymatic conditions assayed. Representative peptides of internal standards (ISs) were also incorporated to ease understanding of targeted quantitative approach regarding normalization of SRM results and analytical quality assessment.

Digestion enzyme	Peptide ID[a]	Retention time (min)[b]	Observed precursor ion (*m/z*)[c]	Peptide charge	Observed SRM fragments (*m/z*)[c]
Trypsin	A2	17.3	1065.1	5+	715.2 (y13++), 954.4 (b36+++), 1203.4 (b22++)
	A1	16.5	1073.0	5+	715.2 (y13 ++), 1311.4 (b36+++), 1429.0 (y13)
	IS1	13.6	493.60		481.6, 639.9, 683.1
	IS2	10.7	718.60		631.4, 744.5, 891.5
Thermolysin	A2	12.6	974.4	2+	1076.8 (y10), 1283.0 (b12), 1735.2 (b16)
	A1	8.7	615.5	2+	565.4 (b5), 665.5 (y6), 1017.9 (b9)
	IS1	8.2	519.1		257.1, 263.2, 501.1
	IS2	7.8	387.0		359.2, 415.3, 599.3
Pepsin	A2	16.3	650.9	2+	430.3 (y4), 794.4 (y8), 871.5 (b8)
	A1	14.7	670.9	2+	539.9 (y10++), 834.5 (y8), 1078.6 (y10)
	IS1	13.3	519.1		257.1, 263.2, 501.1
	IS2	12.2	387.0		359.2, 415.3, 599.3

[a]IS SRM role: IS1, normalizer IS for trypsin and pepsin analyses; quality IS for thermolysin assay. IS2, normalizer IS for thermolysin analysis; quality IS for trypsin and pepsin assays.
[b]Non-matched retention times of ISs regarding thermolysin and pepsin analyses were due to the different chromatographic conditions assayed (see section 2.6).
[c]Considered mass tolerance, ±0.5 Da.

Table S2: Summary of targeted quantitative SRM results of CAL, MIX, and REP batches (see **Figure S1C**) from trypsin-digested (Tryps-) samples. Full details of SRM properties collected in **Table S1**.

CAL BATCH					
**Internal standards**					
**Sample**	**Observed IS (*m/z*)**	**Rt (min)**	**SRM Area[a]**	**Area IS1/IS2[b]**	
**Tryps – CAL 1**	493.6	13.6	2460450	8.58	
	718.6	14.6	286624		
**Tryps – CAL 2**	493.6	13.6	2470500	8.30	
	718.6	14.7	297575		
**Tryps – CAL 3**	493.6	13.7	2005871	7.66	
	718.6	14.7	261816		
**Tryps – CAL 4**	493.6	13.6	3351208	7.28	
	718.6	14.7	460333		
**Tryps – CAL 5**	493.6	13.9	2517510	7.98	
	718.6	15.3	315580		
**Tryps – CAL 6**	493.6	13.7	3074819	7.30	
	718.6	14.8	421433		
**Mean**				7.84	
**Std. Dev**				0.49	
**%CV**				6.2	
**Characteristic A2 precursor ion**					
**Sample**	**µl POOL**	**Observed precursor (*m/z*)**	**Rt (min)**	**SRM Area[a]**	**Normalized area[c]**
**Tryps – CAL 1**	5	1065.1	17.3	221814	0.090
**Tryps – CAL 2**	10	1065.1	17.2	686083	0.278
**Tryps – CAL 3**	20	1065.1	17.3	1278305	0.637
**Tryps – CAL 4**	30	1065.1	17.3	3172408	0.947
**Tryps – CAL 5**	40	1065.1	17.1	3658995	1.453
**Tryps – CAL 6**	50	1065.1	17.3	5511801	1.793
**Characteristic A1 precursor ion**					
**Sample**	**µl POOL**	**Observed percursor (*m/z*)**	**Rt (min)**	**SRM Area[a]**	**Normalized area[c]**
**Tryps – CAL 1**	5	1073.0	16.48	878845	0.357

**Tryps – CAL 2**	10	1073.0	16.53	3007721	1.217
**Tryps – CAL 3**	20	1073.0	16.48	6027825	3.005
**Tryps – CAL 4**	30	1073.0	16.48	17069169	5.093
**Tryps – CAL 5**	40	1073.0	16.12	19759995	7.849
**Tryps – CAL 6**	50	1073.0	16.56	27943963	9.088
**MIX BATCH**					
**Internal standards**					
**Sample**	**Observed IS (*m/z*)**	**Rt (min)**	**SRM Area[a]**	**Area IS1/IS2[b]**	
**Tryps – MIX 1**	493.6	13.58	2841068	7.63	
	718.6	14.62	372258		
**Tryps – MIX 2**	493.6	13.61	2206147	7.22	
	718.6	14.65	305549		
**Tryps – MIX 3**	493.6	13.65	2670715	7.71	
	718.6	14.65	346552		
**Tryps – MIX 4**	493.6	13.61	1786542	7.13	
	718.6	14.68	250737		
**Tryps – MIX 5**	493.6	13.92	2102440	7.09	
	718.6	15.27	296585		
**Mean**				7.35	
**SEM**				0.26	
**%CV**				3.6	
**Characteristic A1 precursor ion**					
**Sample**	**% A1**	**Observed precursor (*m/z*)**	**Rt (min)**	**SRM Area[a]**	**Normalized area[c]**
**Tryps – MIX 1**	0.1	1073.0	16.68	126184	0.044
**Tryps – MIX 2**	0.5	1073.0	16.65	347381	0.157
**Tryps – MIX 3**	1	1073.0	16.53	719069	0.269
**Tryps – MIX 4**	3	1073.0	16.45	1377179	0.771
**Tryps – MIX 5**	5	1073.0	16.62	2523108	1.200
**REP BATCH**					
**Internal standards**					
**Sample**	**Observed IS (*m/z*)**	**Rt (min)**	**SRM Area[a]**	**Area IS1/IS2[b]**	
**Tryps – REP 1**	493.6	13.76	3323402	6.84	
	718.6	14.71	485559		
	493.6	13.67	3382942		

**Tryps – REP 2**	718.6	14.71	519578	6.51
**Tryps – REP 3**	493.6	13.64	3703457	6.72
	718.6	14.67	551334	
**Tryps – REP 4**	493.6	13.7	3671109	7.18
	718.6	14.67	511137	
**Tryps – REP 5**	493.6	13.64	3977000	7.42
	718.6	14.64	535779	
**Tryps – REP 6**	493.6	13.6	3402888	7.39
	718.6	14.6	460705	
**Mean**				7.01
**Std. Dev.**				0.34
**%CV**				4.9
**Characteristic A2 precursor ion**				
**Sample**	**Observed precursor (*m/z*)**	**Rt (min)**	**SRM Area[a]**	**Normalized area[c]**
**Tryps – REP 4**	1065.1	17,37	8016188	2.184
**Tryps – REP 5**	1065.1	17,25	6140185	1.544
**Tryps – REP 6**	1065.1	17,24	7735129	2.273
**Characteristic A1 precursor ion**				
**Sample**	**Observed precursor (*m/z*)**	**Rt (min)**	**SRM Area[a]**	**Normalized area[c]**
**Tryps – REP 1**	1073.0	16,42	34921467	10.508
**Tryps – REP 2**	1073.0	16,53	30637428	9.056
**Tryps – REP 3**	1073.0	16,56	46328098	12.509

Abbreviations used: CAL, calibration; IS, internal standard; MIX, mixture; REP, replicate; Rt, retention time; Std. Dev, standard deviation; %CV, coefficient of variation (%).

[a]Integrated SRM peak areas from FreeStyle analysis (see section 2.9).

[b]IS reliability ratios (IS1 at *m/z* 493.6 and IS2 at *m/z* 718.6 as normalizer and quality ISs, respectively) for robustness assessment of SRM analysis.

[c]Results normalized by IS1 (*m/z* 493.6) according to $Area_{A1-2}/Area_{IS1}$.

Table S3: Summary of targeted quantitative SRM results of CAL, MIX, and REP batches (see **Figure S1C**) from thermolysin-digested (Therm-) samples. Full details of SRM properties collected in **Table S1**.

CAL BATCH					
**Internal standards**					
**Sample**	**Observed IS (*m/z*)**	**Rt (min)**	**SRM Area[a]**	**Area IS1/IS2[b]**	
**Therm – CAL 1**	519.1	8.1	1904598	2.36	
	387.0	7.6	805664		
**Therm – CAL 2**	519.1	8.2	1524149	2.16	
	387.0	7.7	706159		
**Therm – CAL 3**	519.1	8.1	2599563	2.36	
	387.0	7.6	1102676		
**Therm – CAL 4**	519.1	8.2	2782444	2.23	
	387.0	7.7	1246103		
**Therm – CAL 5**	519.1	8.1	3232033	2.38	
	387.0	7.7	1360105		
**Therm – CAL 6**	519.1	8.2	2876733	2.96	
	387.0	7.9	972385		
**Mean**				2.39	
**Std. Dev.**				0.26	
**%CV**				10.8	
**Characteristic A2 precursor ions**					
**Sample**	**µl POOL**	**Observed precursor (*m/z*)**	**Rt (min)**	**SRM Area[a]**	**Normalized area[c]**
**Therm – CAL 1**	5	974.4	12.4	10909	0.014
**Therm – CAL 2**	10	974.4	12.3	28059	0.040
**Therm – CAL 3**	20	974.4	12.3	61663	0.056
**Therm – CAL 4**	30	974.4	12.3	112148	0.090
**Therm – CAL 5**	40	974.4	12.4	159378	0.117
**Therm – CAL 6**	50	974.4	12.4	155996	0.160
**Characteristic A1 precursor ions**					
**Sample**	**µl POOL**	**Observed precursor (*m/z*)**	**Rt (min)**	**SRM Area[a]**	**Normalized area[c]**
**Therm – CAL 1**	5	615.5	8.7	481726	0.598

**Therm – CAL 2**	10	615.5	8.6	1019997	1.444
**Therm – CAL 3**	20	615.5	8.6	2639647	2.394
**Therm – CAL 4**	30	615.5	8.8	4118707	3.305
**Therm – CAL 5**	40	615.5	8.7	6650188	4.889
**Therm – CAL 6**	50	615.5	8.5	6756519	6.948
**MIX BATCH**					
**Internal standards**					
**Sample**	**Observed IS (*m/z*)**	**Rt (min)**	**SRM Area[a]**	**Area IS1/IS2[b]**	
**Therm – MIX 1**	519.1	8.4	2620153	2.70	
	387.0	7.9	972000		
**Therm – MIX 2**	519.1	8.1	3217121	2.72	
	387.0	7.7	1181788		
**Therm – MIX 3**	519.1	8.1	2532428	2.32	
	387.0	7.7	1093244		
**Therm – MIX 4**	519.1	8.1	3237607	2.75	
	387.0	7.7	1177052		
**Therm – MIX 5**	519.1	8.1	2748973	2.51	
	387.0	7.6	1095118		
**Mean**				2.60	
**Std. Dev.**				0.16	
**%CV**				6.3	
**Characteristic A1 precursor ions**					
**Sample**	**% A1 POOL**	**Observed precursor (*m/z*)**	**Rt (min)**	**SRM Area[a]**	**Normalized area[c]**
**Therm – MIX 1**	0.1	615.5	8.7	46709	0.048
**Therm – MIX 2**	0.5	615.5	8.7	94461	0.080
**Therm – MIX 3**	1	615.5	8.6	142049	0.130
**Therm – MIX 4**	3	615.5	8.6	488503	0.415
**Therm – MIX 5**	5	615.5	8.7	745522	0.681
**REP BATCH**					
**Internal standards**					
**Sample**	**Observed IS (*m/z*)**	**Rt (min)**	**SRM Area[a]**	**Area IS1/IS2[b]**	
**Therm – REP 1**	519.1	8.7	4663365	2.77	
	387.0	8.3	1683469		

**Therm – REP 2**	519.1	8.4	3572046	2.54
	387.0	7.9	1407073	
**Therm – REP 3**	519.1	8.2	3956101	2.83
	387.0	7.8	1398155	
**Therm – REP 4**	519.1	8.2	3799912	2.78
	387.0	7.8	1368311	
**Therm – REP 5**	519.1	8.2	3773403	2.63
	387.0	7.8	1433932	
**Therm – REP 6**	519.1	8.1	2907505	2.79
	387.0	7.7	1043534	
**Mean**				2.72
**Std. Dev**				0.10
**%CV**				3.8

**Characteristic A2 precursor ions**				
**Sample**	**Observed precursor (*m/z*)**	**Rt (min)**	**SRM Area[a]**	**Normalized area[c]**
**Therm – REP 4**	974.4	13.0	141972	0.104
**Therm – REP 5**	974.4	13.0	125785	0.088
**Therm – REP 6**	974.4	12.7	164373	0.158
**Characteristic A1 precursor ions**				
**Sample**	**Observed precursor (*m/z*)**	**Rt (min)**	**SRM Area[a]**	**Normalized area[c]**
**Therm – REP 1**	615.5	9.1	11398873	6.771
**Therm – REP 2**	615.5	8.9	9545719	6.784
**Therm – REP 3**	615.5	8.7	15869186	11.350

Abbreviations used: CAL, Calibration; IS, internal standard; MIX, Mixture; REP: Replicate; Rt, retention time; Std. Dev, standard deviation; %CV, coefficient of variation (%).

[a]Integrated SRM peak areas from FreeStyle analysis (see section 2.9).

[b]IS reliability ratios ($Area_{IS1}/Area_{IS2}$, IS1 at *m/z* 519.1 and IS2 at *m/z* 387.0 as quality and normalizer ISs, respectively) for robustness assessment of SRM analysis.

[c]Results normalized by IS2 (*m/z* 387.0) according to $Area_{A1-2}/Area_{IS2}$.

Table S4: Summary of targeted quantitative SRM results of CAL, MIX, and REP batches (see **Figure S1C**) from pepsin-digested (Peps-) samples. Full details of SRM transitions collected in **Table S1**.

CAL BATCH					
**Internal standards**					
**Sample**	**Observed IS (*m/z*)**	**Rt (min)**	**SRM Area[a]**	**Area IS1/IS2[b]**	
**Peps – CAL 1**	519.1	13.9	2889294	2.63	
	387.0	12.5	1096898		
**Peps – CAL 2**	519.1	14.4	2628816	2.67	
	387.0	13.3	984534		
**Peps – CAL 3**	519.1	13.6	2842603	2.19	
	387.0	12.3	1299600		
**Peps – CAL 4**	519.1	13.2	3734038	2.53	
	387.0	12.5	1477780		
**Peps – CAL 5**	519.1	13.0	3281108	2.78	
	387.0	12.4	1179590		
**Peps – CAL 6**	519.1	13.4	3317236	2.57	
	387.0	12.3	1289731		
**Mean**				2.56	
**Std. Dev**				0.19	
**%CV**				7.2	
**Characteristic A2 precursor ions**					
**Sample**	**µl POOL**	**Observed precursor**	**Rt (min)**	**SRM Area[a]**	**Normalized area[c]**
**Peps – CAL 1**	5	650.9	16.44	281775	0.098
**Peps – CAL 2**	10	650.9	16.42	699117	0.266
**Peps – CAL 3**	20	650.9	15.75	2256142	0.794
**Peps – CAL 4**	30	650.9	16.43	4639715	1.243
**Peps – CAL 5**	40	650.9	16.54	5668945	1.728
**Peps – CAL 6**	50	650.9	16.3	8091527	2.439
**Characteristic A1 precursor ions**					
**Sample**	**µl POOL**	**Observed precursor (*m/z*)**	**Rt (min)**	**SRM Area[a]**	**Normalized area[c]**

**Peps – CAL 1**	5	670.9	14.2	1109590	0.384
**Peps – CAL 2**	10	670.9	14.8	2738145	1.042
**Peps – CAL 3**	20	670.9	15.1	7988957	2.810
**Peps – CAL 4**	30	670.9	14.7	17246457	4.619
**Peps – CAL 5**	40	670.9	14.7	20842013	6.352
**Peps – CAL 6**	50	670.9	14.9	26957098	8.126
**MIX BATCH**					
**Internal standards**					
**Sample**	**Observed IS (*m/z*)**	**Rt (min)**	**SRM Area[a]**	**Area IS1/IS2[b]**	
**Peps – MIX 1**	519.1	12.9	4141929	2.56	
	387.0	11.8	1621012		
**Peps – MIX 2**	519.1	13.2	4209341	2.33	
	387.0	12.0	1803960		
**Peps – MIX 3**	519.1	13.3	2553727	2.93	
	387.0	13.0	872930		
**Peps – MIX 4**	519.1	14.4	2375424	2.29	
	387.0	12.4	1035629		
**Peps – MIX 5**	519.1	13.4	3767297	2.47	
	387.0	12.4	1523060		
**Mean**				2.51	
**Std. Dev.**				0.23	
**%CV**				9.1	
**Characteristic A1 precursor ions**					
**Sample**	**% A1**	**Observed precursor**	**Rt (min)**	**SRM Area[a]**	**Normalized area[c]**
**Peps – MIX 1**	0.1	670.9	14.7	186201	0.045
**Peps – MIX 2**	0.5	670.9	15.6	551952	0.131
**Peps – MIX 3**	1	670.9	14.4	457784	0.179
**Peps – MIX 4**	3	670.9	14.6	2178545	0.917
**Peps – MIX 5**	5	670.9	14.9	4044580	1.074
**REP BATCH**					
**Internal standards**					
**Sample**	**IS**	**RT**	**SRM Area[a]**	**Area IS1/IS2[b]**	
**Peps – REP 1**	519.1	12.8	4721211	2.63	
	387.0	11.7	1798130		

**Peps – REP 2**	519.1	12.9	5949930	2.54	
	387.0	11.7	2345777		
**Peps – REP 3**	519.1	12.9	4616176	2.57	
	387.0	11.7	1796711		
**Peps – REP 4**	519.1	13.0	3907614	2.64	
	387.0	11.8	1480335		
**Peps – REP 5**	519.1	12.8	4244428	2.58	
	387.0	11.7	1645508		
**Peps – REP 6**	519.1	12.9	5035658	2.51	
	387.0	11.7	2004782		
**Mean**				2.58	
**SEM**				0.05	
**%CV**				1.8	

**Characteristic A2 precursor ions**				
**Sample**	**Observed precursor (*m/z*)**	**Rt (min)**	**SRM Area[a]**	**Normalized area[c]**
**Peps – REP 4**	650.9	16.4	10567533	2.704
**Peps – REP 5**	650.9	16.4	12361761	2.912
**Peps – REP 6**	650.9	16.4	17306317	3.437
**Characteristic A1 precursor ions**				
**Sample**	**Observed precursor (*m/z*)**	**Rt (min)**	**SRM Area[a]**	**Normalized area[c]**
**Peps – REP 1**	670.9	14.6	37205027	7.880
**Peps – REP 2**	670.9	14.6	54511351	9.162
**Peps – REP 3**	670.9	14.6	49299499	10.680

Abbreviations used: CAL, calibration; IS, internal standard; MIX, mixture; REP: Replicate; Rt, retention time; Std. Dev, standard deviation; %CV, coefficient of variation (%).
[a]Integrated SRM peak areas from FreeStyle analysis (see section 2.9).
[b]IS reliability ratios (IS1 at *m/z* 519.1 and IS2 at *m/z* 387.0 as normalizer and quality ISs, respectively) for robustness assessment of SRM analysis.
[c]Results normalized by IS1 (*m/z* 519.1) according to $Area_{A1-2}/Area_{IS1}$.

Table S5. Summary of targeted quantitative SRM results of QC batches from trypsin-, thermolysin- and pepsin-digested samples. Full details of SRM transitions collected in **Table S1.**

**QC BATCH-Trypsin digestion**				
**Internal standards**				
**Sample**	**Observed IS (*m/z*)**	**Rt (min)**	**SRM Area[a]**	**Area IS1/IS2[b]**
**QC1**	493.6	13.74	3562672	7.20
	718.6	14.64	494860	
**QC2**	493.6	13.64	2363258	6.49
	718.6	14.67	364255	
**QC3**	493.6	13.54	2361977	7.29
	718.6	14.54	324091	
**Mean**				6.99
**Std. Dev.**				0.36
**%CV**				5.1
**Characteristic A2 precursor ion**				
**Sample**	**Observed Peptide (*m/z*)**	**Rt (min)**	**SRM Area[a]**	**Normalized area[c]**
**QC1**	1065.1	17.27	4096413	1.15
**QC2**	1065.1	17.20	2856665	1.21
**QC3**	1065.1	17.24	2617235	1.11
**Mean**				1.16
**Std. Dev.**				0.04
**%CV**				3.6
**Characteristic A1 precursor ion**				
**Sample**	**Observed Peptide (*m/z*)**	**Rt (min)**	**SRM Area[a]**	**Normalized area[c] (519.1)**
**QC1**	1073.0	16.43	20203362	5.671
**QC2**	1073.0	16.52	14110773	5.971
**QC3**	1073.0	16.53	12532241	5.306
**Mean**				5.64
**Desvest**				0.27
**%CV**				4.8

Abbreviations used: QC, quality control; IS, internal standard; Rt, retention time; Std. Dev, standard deviation; %CV, coefficient of variation (%).
[a]Integrated SRM peak areas from FreeStyle analysis (see section 2.9).
[b]IS reliability ratios (IS1 at *m/z* 493.6 and IS2 at *m/z* 718.6 as normalizer and quality ISs, respectively) for robustness assessment of SRM analysis.
[c]Results normalized by IS1 (*m/z* 493.6) according to $Area_{A1-2}/Area_{IS1}$.

Table S5 (continuation)

QC BATCH-Thermolysin digestion				
**Internal standards**				
**Sample**	**Observed IS (*m/z*)**	**Rt (min)**	**SRM Area[a]**	**Area IS1/IS2[b]**
**QC1**	519.1	8.26	1367028	2.88
	387.0	7.65	475126	
**QC2**	519.1	7.21	1257869	2.50
	387.0	6.31	503590	
**QC3**	519.1	7.12	1221268	2.40
	387.0	6.14	508299	
**Mean**				2.59
**Std. Dev.**				0.21
**%CV**				8.0
**Characteristic A2 precursor ions**				
**Sample**	**Observed peptide (*m/z*)**	**Rt (min)**	**SRM Area[a]**	**Normalized area[c]**
**QC1**	974.4	12.31	104041	0.22
**QC2**	974.4	12.06	86817	0.17
**QC3**	974.4	12.21	106167	0.21
**Mean**				0.20
**Std. Dev.**				0.02
**%CV**				10.9
**Characteristic A1 precursor ions**				
**Sample**	**Observed Peptide (*m/z*)**	**Rt (min)**	**SRM Area[a]**	**Normalized area[c]**
**QC1**	615.5	8.34	3740841	7.87
**QC2**	615.5	8.16	3888812	7.72
**QC3**	615.5	8.05	3912875	7.70
**Mean**				7.76
**Std. Dev.**				0.08
**%CV**				1.0

Abbreviations used: QC, quality control; IS, internal standard; Rt, retention time; Std. Dev, standard deviation of the mean; %CV, coefficient of variation (%).

[a]Integrated SRM peak areas from FreeStyle analysis (see section 2.9).

[b]IS reliability ratios ($Area_{IS1}/Area_{IS2}$, IS1 at *m/z* 519.1 and IS2 at *m/z* 387.0 as quality and normalizer ISs, respectively) for robustness assessment of SRM analysis.

[c]Results normalized by IS2 (*m/z* 387.0) according to $Area_{A1\text{-}2}/Area_{IS2}$.

Table S5 (continuation)

QC BATCH-Pepsin digestion				
**Internal standards**				
**Sample**	**Observed IS (*m/z*)**	**Rt (min)**	**SRM Area[a]**	**Area IS1/IS2[b]**
**QC1**	519.1	13.31	2769847	2.60
	387.0	12.35	1066885	
**QC2**	519.1	13.40	2527947	2.46
	387.0	12.33	1028951	
**QC3**	519.1	13.69	2649073	2.98
	387.0	12.98	888984	
**Mean**				2.67
**Std. Dev.**				0.22
**%CV**				8.2
**Characteristic A2 precursor ions**				
**Sample**	**Observed Peptide (m/z) (m/z)**	**Rt (min)**	**SRM Area[a]**	**Normalized area[c]**
**QC1**	650.9	16.57	4646297	1.68
**QC2**	650.9	16.18	3978484	1.57
**QC3**	650.9	16.65	4094335	1.55
**Mean**				1.60
**Std. Dev.**				0.06
**%CV**				3.6
**Characteristic A1 precursor ions**				
**Sample**	**Observed Peptide (*m/z*)**	**Rt (min)**	**SRM Area[a]**	**Normalized area[c]**
**QC1**	670.9	14.76	17532801	6.33
**QC2**	670.9	15.72	14109462	5.58
**QC3**	670.9	14.99	15052098	5.68
**Mean**		15.16	15564787	5.85
**Std. Dev.**		0.50	1768318	0.33
**%CV**				5.7

Abbreviations used: QC, quality control; IS, internal standard; Rt, retention time; Std. Dev, standard deviation of the mean; %CV, coefficient of variation (%).

[a]Integrated SRM peak areas from FreeStyle analysis (see section 2.9).

[b]IS reliability ratios (IS1 at *m/z* 519.1 and IS2 at *m/z* 387.0 as normalizer and quality ISs, respectively) for robustness assessment of SRM analysis.

[c]Results normalized by IS1 (*m/z* 519.1) according to $Area_{A1-2}/Area_{IS1}$.

Table S6: Summary of internal standard reliability ratios yielded by targeted quantitative SRM analyses for CAL, MIX, REP and QC batches considering all enzymatic conditions assayed.

SAMPLE	Trypsin	Thermolysin	Pepsin
	Area IS1/IS2[a]	Area IS1/IS2[b]	Area IS1/IS2[b]
**Cal 1**	8.58	2.36	2.63
**Cal 2**	8.30	2.16	2.67
**Cal 3**	7.66	2.36	2.19
**Cal 4**	7.28	2.23	2.53
**Cal 5**	7.98	2.38	2.78
**Cal 6**	7.30	2.96	2.57
**Mix 1**	7.63	2.70	2.56
**Mix 2**	7.22	2.72	2.33
**Mix 3**	7.71	2.32	2.93
**Mix 4**	7.13	2.75	2.29
**Mix 5**	7.09	2.51	2.47
**Rep 1**	6.84	2.77	2.63
**Rep 2**	6.51	2.54	2.54
**Rep 3**	6.72	2.83	2.57
**Rep 4**	7.18	2.78	2.64
**Rep 5**	7.42	2.63	2.58
**Rep 6**	7.39	2.79	2.51
**QC 1**	7.20	2.88	2.60
**QC 2**	6.49	2.50	2.46
**QC 3**	7.29	2.40	2.98
**Mean**	**7.35**	**2.58**	**2.57**
**Std. Dev.**	**0.53**	**0.23**	**0.19**
**% CV**	**7.28**	**9.02**	**7.30**

Abbreviations used: IS, internal standard; CAL, calibration; MIX, mixture; REP: Replicate; QC, quality control; Std. Dev, standard deviation of the mean; %CV, coefficient of variation (%).

[a] IS reliability ratios (IS1 at *m/z* 493.6 and IS2 at *m/z* 718.6) for robustness assessment of SRM analysis.

[b] IS reliability ratios (IS1 at *m/z* 519.1 and IS2 at *m/z* 387.0) for robustness assessment of SRM analysis.
